# SAIBR: a simple, platform-independent method for spectral autofluorescence correction

**DOI:** 10.1242/dev.200545

**Published:** 2022-07-14

**Authors:** Nelio T. L. Rodrigues, Tom Bland, Joana Borrego-Pinto, KangBo Ng, Nisha Hirani, Ying Gu, Sherman Foo, Nathan W. Goehring

**Affiliations:** 1 Francis Crick Institute, London NW1 1AT, UK; 2 Institute for the Physics of Living Systems, University College London, London WC1E 6BT, UK; 3 Randall Centre for Cell and Molecular Biophysics, School of Basic and Medical Biosciences, King's College London, London SE1 1UL, UK

**Keywords:** Autofluorescence correction, *C. elegans*, Starfish, *S. pombe*, Fiji plug-in

## Abstract

Biological systems are increasingly viewed through a quantitative lens that demands accurate measures of gene expression and local protein concentrations. CRISPR/Cas9 gene tagging has enabled increased use of fluorescence to monitor proteins at or near endogenous levels under native regulatory control. However, owing to typically lower expression levels, experiments using endogenously tagged genes run into limits imposed by autofluorescence (AF). AF is often a particular challenge in wavelengths occupied by commonly used fluorescent proteins (GFP, mNeonGreen). Stimulated by our work in *C. elegans*, we describe and validate Spectral Autofluorescence Image Correction By Regression (SAIBR), a simple platform-independent protocol and FIJI plug-in to correct for autofluorescence using standard filter sets and illumination conditions. Validated for use in *C. elegans* embryos, starfish oocytes and fission yeast, SAIBR is ideal for samples with a single dominant AF source; it achieves accurate quantitation of fluorophore signal, and enables reliable detection and quantification of even weakly expressed proteins. Thus, SAIBR provides a highly accessible low-barrier way to incorporate AF correction as standard for researchers working on a broad variety of cell and developmental systems.

## Abstract (precis)

**Summary:** Implemented as an easy-to-use Fiji plug-in, SAIBR provides effective autofluorescence correction for cells and tissues using standard off-the-shelf imaging conditions.

## INTRODUCTION

Owing to its highly reproducible development and simple geometry, *C. elegans* has emerged as an ideal system for quantitative analysis of symmetry-breaking (Gross et al., 2019; Lang and Munro, 2017), cell division (Pintard and Bowerman, 2019), cell and tissue mechanics (Zhang et al., 2010), and cellular decision making (Barkoulas et al., 2013). Furthermore, there is a wealth of endogenously tagged genes of interest allowing live quantitative imaging of protein networks operating at native expression levels (Dickinson and Goldstein, 2016). However, despite being transparent, *C. elegans* exhibits significant intrinsic autofluorescence (AF) produced by a variety of cellular constituents, and AF can be observed across all stages of worm development (Croce and Bottiroli, 2014; Pincus et al., 2016). It is most prominent when using blue and ultraviolet excitation wavelengths, and thus poses problems when using standard GFP illumination conditions (Heppert et al., 2016). Consequently, there is a need for efficient and easily implemented methods for AF correction.

A number of general strategies have been sought to correct for AF. One approach is to experimentally suppress AF. One can use chemical compounds to specifically reduce or quench AF background, or even pre-bleach samples before fluorophore addition, although these methods tend to be restricted to fixed samples (Billinton and Knight, 2001; Cowen et al., 1985; Neumann and Gabel, 2002). In *C. elegans*, *glo* mutants exhibit reduced formation of autofluorescent gut granules, although their abnormal physiology may complicate analysis (Hermann et al., 2005).

Another approach has been to optimize the combination of fluorophores, excitation light sources and emission filters to maximize the separation between fluorophore signal and AF (Billinton and Knight, 2001). These strategies usually require specialized imaging setups, and narrow emission bands can often limit the signal being captured. Such approaches may also restrict the choice of fluorophores. In *C. elegans*, the overlap of the excitation and emission spectra of AF with commonly used green fluorescence proteins, such as GFP or mNeonGreen (mNG), makes this approach difficult to achieve in practice. Nevertheless, some success has been achieved with specialized filter sets (An and Blackwell, 2003; Teuscher and Ewald, 2018) or yellow-shifted excitation, which is compatible with mNG, but avoids the AF excitation peak (Heppert et al., 2016). One can also avoid the AF excitation peak in *C. elegans* by using red-shifted RFPs such as mCherry and mKate2. However, compared with GFP and mNG, red fluorophores are less optimal due to reduced quantum yield, lower brightness and enhanced photobleaching (Heppert et al., 2016; Shaner et al., 2004; Shcherbo et al., 2009).

Techniques such as fluorescence lifetime and spectral imaging can resolve overlapping fluorescent signals based on their distinct fluorescent lifetimes or spectral characteristics (Billinton and Knight, 2001; Kumar et al., 2009; Mansfield et al., 2005). AF typically exhibits fluorescence characteristics that are distinct from other fluorophores and, therefore, it can often be separated out much as one would an additional fluorophore. Such approaches have been useful in compensating for the high levels of gut autofluorescence in *C. elegans* (Shi and Grant, 2015). However, such techniques require specialized instruments and analytical tools, to which some may not have access or which may be incompatible with particular experimental workflows.

An alternative, but related approach to spectral unmixing is so-called ‘AF subtraction’ (Alberti et al., 1987; Billinton and Knight, 2001; Steinkamp and Stewart, 1986). First proposed for flow cytometry, this rather simple methodology takes advantage of the fact that AF typically exhibits much broader emission spectra than fluorophores such as GFP. One can therefore quantify AF in a given sample using an AF-reporting channel and, with the appropriate calibration, subtract it on a pixel-by-pixel basis from the signal measured in the fluorophore channel (Pang et al., 2013; Roederer, 2002; Van de Lest et al., 1995). The advantages of this approach are that it is relatively straightforward to implement, it uses commonly available light sources and excitation/emission filters, and it does not require significant prior knowledge about fluorescence spectra beyond identifying a channel that is relatively specific for AF. Variations of this basic technique have since been applied in a variety of contexts, including cell-based monitoring of gene expression and AF correction of fluorescence *in situ* hybridization samples (Chen et al., 2019; Davis et al., 2010; Lichten et al., 2014; Roederer and Murphy, 1986; Szöllösi et al., 1995).

Here, we demonstrate that AF subtraction is a powerful method for autofluorescence correction during fluorescence imaging of *C. elegans* embryos. Notably, our implementation achieves results on par with more specialized imaging modalities, correcting both for bulk whole embryo fluorescence as well as for spatial AF variation. It enables reliable optimization of fluorescence signal from even very weakly expressed endogenous GFP fusions, and it is compatible with dual labeled GFP/mCherry samples, bringing substantial improvements to fluorescence signal quantification. At the same time, owing to its use of standard GFP/RFP filters sets and its implementation via an easy-to-use Fiji plug-in, this protocol, which we term Spectral Autofluorescence Image correction By Regression (SAIBR), is readily combined with a variety of imaging platforms and procedures, allowing integration of AF correction as a standard part of imaging workflows. Although developed with *C. elegans* embryos in mind, the principles behind SAIBR are general and thus should be suitable for a variety of samples and fluorophores. Consistent with this, our SAIBR plug-in is readily applicable to a variety of other experimental systems, and thus should be a useful tool for AF correction for the cell and developmental biology community.

## RESULTS

**Fig. 1.**
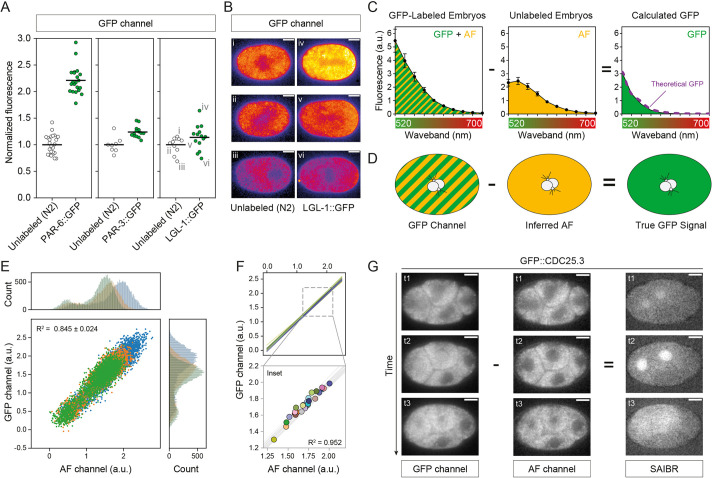
**Autofluorescence in *C. elegans* is correlated across a broad spectrum of wavelengths.** (A) Normalized fluorescence intensity for embryos expressing PAR-6::GFP, PAR-3::GFP or LGL::GFP (strains: KK1248, KK1216 and NWG0285, respectively) relative to unlabeled N2 embryo controls reveals that AF contributes substantially to total measured fluorescence signal. Data for individual embryos are shown with mean indicated. (B) GFP channel images of unlabeled N2 and LGL-1::GFP embryos captured using identical parameters. Embryos are taken from dataset in A. (C) Subtraction of the fluorescence emission spectrum of unlabeled embryos (AF signal) from that of GFP::PAR-6 expressing embryos yields a calculated GFP spectrum that is indistinguishable from the expected theoretical GFP spectrum (purple dashed line) demonstrating AF and GFP signals are additive. Fluorescence emission from embryos was measured in 20 nm wavebands with midpoints from 520 to 700 nm. Data are mean±s.d. N2 (unlabeled), five embryos; PAR-6::GFP, five embryos. (D) Schematic of the SAIBR approach. Raw GFP channel images consist of both GFP and AF signal. True GFP signal is obtained by subtracting inferred AF signal in the GFP channel derived from AF measurement in a second channel. (E) Individual pixel values are well correlated between Gaussian-filtered (radius=1) AF and GFP channels, and are similar between embryos. Pixels from a region of interest comprising the entire embryo and a small section of surrounding background were used for the regression fit. A random selection comprising 10% of all pixels is shown color-coded per embryo. Histograms of intensity values for GFP and AF channels are shown for reference. (F) Comparison of per-pixel correlation with data obtained from whole embryo means. Lines indicate per-pixel regression from individual embryos as in E. An overlay of mean whole-embryo fluorescence values (circles) is shown below. (G) Application of SAIBR to a GFP::CDC-25.3-expressing embryo (DG4190). Subtraction of inferred AF signal reveals nuclear accumulation beginning at the start of the four-cell stage, and protein release at NEBD (t3). Scale bars: 10 µm.

To quantify the potential impact of AF on the specific detection of GFP in *C. elegans* embryos, we began by comparing the magnitude of AF signal obtained from unlabeled embryos with the signal obtained from embryos expressing GFP fusion proteins when imaged with standard GFP illumination settings (ex^488^/em^535/50^, hereafter GFP channel). For this purpose, we selected *C. elegans* lines expressing GFP fusions to one of three polarity proteins: PAR-6 and PAR-3, which localize to an anterior plasma membrane domain; and LGL-1, which localizes to the posterior plasma membrane. All genes were tagged at the endogenous loci. In these cases, AF accounted for ∼40-90% of the observed signal in the GFP emission band (Fig. 1A). Moreover, when we imaged unlabeled embryos, the AF signal in the GFP channel was intrinsically variable. Not only was there substantial spatial variation in AF (Fig. 1B), but also a nearly twofold variation in the overall magnitude of AF signal between embryos (Fig. 1A,B, embryo i versus iii). Thus, simply subtracting out mean AF signal obtained from unlabeled embryos will fail to account for both sources of variation. In the case of LGL-1::GFP, such mean AF subtraction would clearly lead to apparent negative concentrations in some embryos. Thus, if we wish to accurately quantify the expression and local subcellular concentrations of proteins using GFP fusions, particularly for genes exhibiting low-to-moderate expression, one requires a method for locally correcting AF on a per embryo, per pixel basis.

### Implementing a simplified method for AF correction based on dual emission imaging

In principle, the emission signal for GFP and AF will be additive; thus, if one has an independent measure of AF, one can subtract AF from the combined signal to obtain a value for GFP emission. Indeed, if we subtract the fluorescence emission spectrum of autofluorescence measured in unlabeled embryos from the spectrum obtained from embryos expressing PAR-6::GFP, we almost perfectly recover the theoretical spectrum for GFP (Fig. 1C). The challenge is therefore to find a method of quantifying AF directly in embryos also expressing GFP.

One strategy for quantifying AF takes advantage of the distinct spectral properties of AF that allow it to be quantified in an AF-reporting channel distinct from that used for measuring GFP, hereafter AF or ‘predictor’ channel (Alberti et al., 1987; Roederer and Murphy, 1986). One can use AF channel measurements to predict and thus correct for AF in the ‘primary’ GFP channel (Fig. 1D).

In *C. elegans* embryos, AF peaks in the green-to-yellow wavelengths overlapping GFP, but extending further into the red (Fig. 1C) (Heppert et al., 2016; Pincus et al., 2016). It can therefore be captured on a relatively selective basis through the use of a suitably red-shifted emission filter, such as those typically used for red fluorescent proteins. We therefore specify the AF Channel as ex^488^/em^630/75^. In practice, there will be a slight spillover of GFP signal into the AF channel, which could lead to overestimation of AF; however, because spillover is necessarily proportional to GFP amounts, it can be easily accounted for (see Materials and Methods). To establish an AF correction function between channels, we performed a linear regression on fluorescence pixel values obtained from images of unlabeled embryos captured in both the GFP and AF channels, where all signal is attributable to AF. We obtained strong linear correlations (R^2^>0.8) that were similar between embryos (Fig. 1E). A nearly identical correlation was observed when we plotted the mean intensity values of entire individual embryos (R^2^=0.952, Fig. 1F), indicating that the same correction function can account for both intra- and inter-embryo AF variation. Thus, we can use AF measurement in the AF channel to accurately infer and subtract AF signal from the GFP channel to obtain an accurate measure of ‘true GFP’ signal (Fig. 1D).

**Fig. 2.**
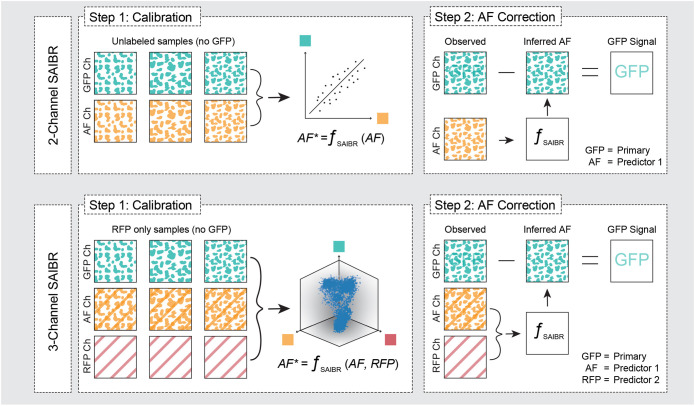
**Schematic summary of SAIBR workflow.** The first step for two-channel SAIBR is to define the correction function ƒ_SAIBR_. ƒ_SAIBR_ is defined by imaging multiple unlabeled calibration samples in which all fluorescence signal arises from autofluorescence, and performing a linear regression on fluorescence values in the GFP and AF channels. In step 2, to isolate GFP signal in GFP-labeled samples, measured AF channel signal is used to infer AF signal (AF*) in the GFP channel using ƒ_SAIBR_, and inferred AF signal is then subtracted from the observed GFP channel signal to yield the ‘true GFP’ signal. In three-channel SAIBR, one must compensate for red fluorophore (e.g. RFP) signal bleedthrough into the AF channel. Therefore, for three-channel SAIBR, ƒ_SAIBR_ is defined by imaging samples expressing only RFP and performing a multiple linear regression to correlate observed AF and RFP channel signals to signal in the GFP channel. ƒ_SAIBR_ can then be used to infer AF in GFP-labeled samples based on AF and RFP channel signal, which can then be subtracted from the from observed GFP channel signal to obtain the true GFP signal.

As proof of principle, we captured images of embryos expressing a GFP fusion to CDC-25.3 from the endogenous locus. CDC-25.3 expression is repressed until early embryogenesis, reportedly becoming visible as embryos progress beyond the eight-cell stage (Tsukamoto et al., 2017). Using our AF correction method, we were able to observe clear nuclear localization already at the start of the four-cell stage and accurately track its accumulation and release at NEBD at a time at which AF almost completely masked its expression in uncorrected images (Fig. 1G). We designated this protocol Spectral Autofluorescence Image Correction By Regression (SAIBR). A schematic workflow is provided in Fig. 2, with additional details in the Materials and Methods.

### AF correction using SAIBR

**Fig. 3.**
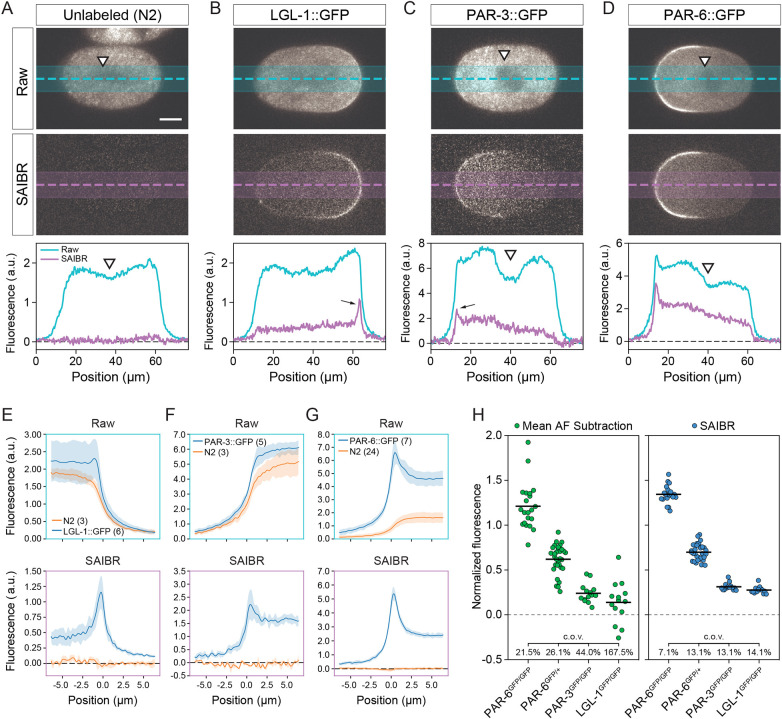
**Quantitative analysis of GFP concentrations by SAIBR.** (A-D) Raw (top) and SAIBR-corrected (middle) midplane images of zygotes expressing the indicated GFP fusions imaged in the GFP channel as shown, along with an associated fluorescence linescan taken as indicated (bottom). SAIBR reduces GFP channel signal to background in unlabeled embryos (A) and reveals a prominent membrane-localized signal (arrows, B,C) that, before correction, is only obvious in PAR-6::GFP embryos. The prominent depletion of signal in the pronuclear/spindle regions in GFP channel images (white arrowheads) largely disappears in SAIBR images, suggesting it is due to local exclusion of AF signal. Strains are KK1216, KK1248 and NWG0285. (E-G) Averaged membrane profiles taken from either raw (top) or SAIBR-corrected (bottom) images of for LGL-1::GFP (E), PAR-3::GFP (F) and PAR-6::GFP (G) shown relative to unlabeled N2 controls. Plasma membrane position is defined as x=0 µm. Data are mean±s.d. Number of embryos is indicated (parentheses). Membrane peaks are strongly enhanced for each GFP fusion-expressing embryo, while N2 profiles are reduced to background in all datasets. (H) Quantitation of mean embryo GFP signal after correction by either mean AF subtraction (green) or SAIBR (blue) for embryos expressing PAR-6::GFP in single (PAR-6^GFP/+^) or double copy (PAR-6^GFP/GFP^), PAR-3::GFP (PAR-3^GFP/GFP^) or LGL-1::GFP (LGL-1^GFP/GFP^). Mean fluorescence values for individual embryos shown with group mean and coefficient of variation (c.o.v.) indicated. Strains used are N2, KK1248, KK1216 and NWG0285. Scale bar: 10 µm.

We next undertook a detailed quantitative analysis of the effectiveness of SAIBR in both unlabeled and GFP-labeled embryos, using GFP as the primary and AF as the predictor channels. Applying SAIBR to unlabeled embryos effectively reduced observed embryo fluorescence in the GFP channel to background, suggesting we accounted for nearly all AF signal in zygotes (Fig. 3A). We then applied SAIBR to embryos expressing GFP fusions to LGL-1, PAR-3 or PAR-6 from the respective endogenous loci (Fig. 3B-D). For both LGL-1::GFP and PAR-3::GFP, SAIBR revealed a clear peak in signal at the posterior and anterior plasma membranes, respectively, that was obscured by AF in uncorrected images (Fig. 3B,C). Even when averaging cross-sectional membrane profiles across multiple embryos, membrane signal was difficult to discern in uncorrected data (Fig. 3E,F, top). By contrast, SAIBR resolved membrane signal into a clear, well-defined peak (Fig. 3E,F, bottom). Cytoplasmic signal also became substantially more uniform, which was most clearly visible in the suppression of a local fluorescence minimum in the embryo center due to AF exclusion by the pronuclei and mitotic spindle region. Improvements are also visible for PAR-6::GFP-expressing embryos, although the magnitude of improvement is less striking due to the higher ratio of GFP to AF signal (Fig. 3D,G). Similar results were achieved using both spinning disk confocal (Fig. 3) and wide-field microscopy (Fig. S1), confirming that the method is platform independent.

We next turned to quantification of total protein concentrations in embryos and compared SAIBR with a mean AF subtraction protocol (mean AF subtraction). For mean AF subtraction, we establish a mean AF signal in the GFP channel based on fluorescence signal measured across multiple unlabeled embryos not expressing GFP and simply subtracted this value from the GFP channel signal in GFP-expressing embryos. As a test, we used *C. elegans* lines expressing GFP::PAR proteins described above as well as embryos that are heterozygous for the *par-6::gfp* fusion and thus only half of the PAR-6 pool is labeled. For PAR-6::GFP, both SAIBR and mean AF subtraction yield the expected 2:1 ratio of GFP signal between homozygous and heterozygous embryos (Fig. 3H). However, by correcting for embryo-to-embryo AF variation, SAIBR substantially reduced the coefficient of variation (c.o.v.). We obtained similar results for PAR-3 and LGL-1 (Fig. 3H). For LGL-1, in which the GFP signal was of the same order as variation in AF, the advantage of SAIBR was particularly striking. Whereas correction by mean AF subtraction resulted in negative values for GFP in embryos, the ability of SAIBR to suppress the effects of embryo-to-embryo variation in AF allowed it to achieve consistent and positive values for GFP signal in all embryos.

### Benchmarking against alternative strategies

**Fig. 4.**
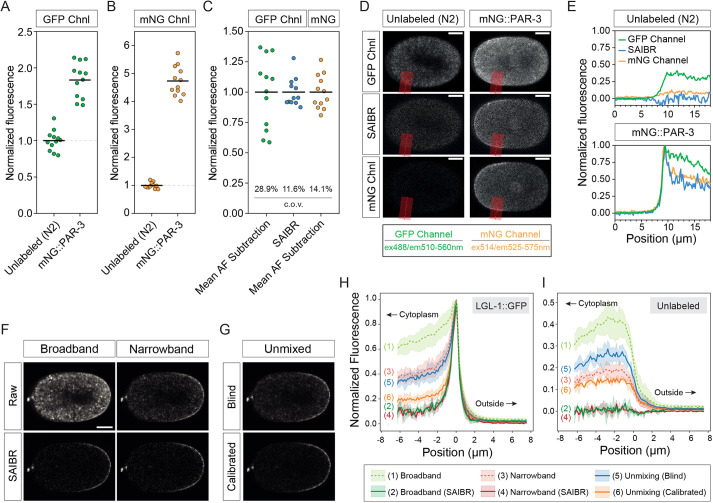
**SAIBR achieves similar results to more specialized AF correction regimes.** (A) Raw fluorescence signal using GFP illumination (GFP channel, ex^488^/em^510-560^) for unlabeled (N2) or PAR-3::mNG embryos (strain is NWG0189). Mean values are indicated. (B) As in A for mNG-specific illumination (mNG channel, ex^514^/em^525-575^). (C) Comparison of mean normalized mNG fluorescence under three AF correction regimes, as indicated. SAIBR and mNG-specific illumination yield similar reductions in the coefficient of variation (c.o.v.) relative to standard GFP illumination with mean AF subtraction. (D) Midplane images of excited embryos imaged using the GFP channel or mNG channel, and either left unprocessed (raw) or subjected to SAIBR, as indicated. PAR-3::mNG and unlabeled N2 are shown for comparison. Scale bars: 10 µm. (E) Plots of profiles taken perpendicularly across the plasma membrane (along the red line in embryos in D) highlight similar performance of SAIBR and use of the mNG channel in reducing AF. (F) Midplane images of a single LGL-1::GFP embryo captured via narrow (ex^488^/em^504-512^) or broad (ex^488^/em^504-557^) emission bands shown either uncorrected or corrected via SAIBR. (G) Same embryo as in F corrected for AF via spectral unmixing using either reference-free (blind) or reference-calibrated (calibrated) automatic component extraction. Images in F and G are individually normalized to 0.01% saturated pixels. (H,I) Plots of a 16 µm profile taken perpendicularly across the plasma membrane and averaged over a domain spanning the posterior 30% for LGL-1::GFP (H) and unlabeled (I) embryos. Fluorescence normalized to respective peak LGL-1::GFP signal. Plasma membrane position is defined as x=0. Regions outside the embryo, the cytoplasm and number references to the appropriate imaging conditions are indicated in the legend below. Data are mean±95% CI [computed by bootstrapping, *n*=5 (LGL::1GFP), *n*=3 (unlabeled)].

To benchmark our method with other approaches, we used an alternative AF-minimization strategy in which we use a fluorophore compatible with wavelengths that minimize AF excitation. mNG behaves similarly to GFP under standard GFP illumination settings (488 nm). However, owing to a slight shift in its excitation spectrum, unlike GFP, it can be efficiently excited by a yellow-shifted laser line (514 nm) to substantially reduce AF (hereafter, *mNG Channel*, ex^514^/em^550/50^) (Heppert et al., 2016). Consistent with this observation, the magnitude of AF signal, as measured in unlabeled embryos relative to total signal for embryos expressing mNG::PAR-3, is reduced substantially in the mNG channel relative to the GFP channel (compare Fig. 4A with 4B). We next compared the effectiveness of AF correction in three regimes: a standard regime using the GFP channel and mean AF subtraction, as described in the previous section; an mNG-specific regime using the mNG channel and mean AF subtraction; and a regime using the GFP channel but corrected by SAIBR. By plotting normalized corrected signal, we found that using either the mNG channel or SAIBR regimes showed similar and substantial improvement in the variance of mean embryo fluorescence (Fig. 4C), suppression of spatially varying cytoplasmic AF (Fig. 4D) and accurate quantification of membrane signal (Fig. 4E).

Using LGL-1::GFP as a case study, we also compared SAIBR with several AF suppression tools available as ‘off-the-shelf’ tools on the Zeiss 880, including using an optimized emission band (499-508 nm) and spectral unmixing. Spectral unmixing uses a built-in automatic component extraction algorithm, which can be performed in both reference-free (‘blind’) and reference-calibrated (‘calibrated’) modes. All three methods substantially reduced AF compared with images captured using typical broadband GFP emission band (499-562 nm), which served as a reference (Fig. 4F-I). Unsurprisingly, of the three, calibrated spectral unmixing showed the best performance when judged by either membrane-to-cytoplasm ratios in LGL-1::GFP-expressing animals or by their ability to reduce AF in unlabeled embryos (Fig. 4H,I). However, SAIBR generally outperformed all three methods for our samples, reducing AF effectively to zero in unlabeled embryos and achieving the highest membrane:cytoplasm ratios for LGL-1::GFP. Results were similar for both narrowband and broadband emission, despite the increased AF present in the latter images. Thus, SAIBR is highly competitive with other state-of-the-art AF compensation techniques, providing comparable and, in some cases, better improvement in image quality and signal quantitation without the need for specialized imaging modalities typically required for other techniques.

### Extension of SAIBR to dual-labeled samples

**Fig. 5.**
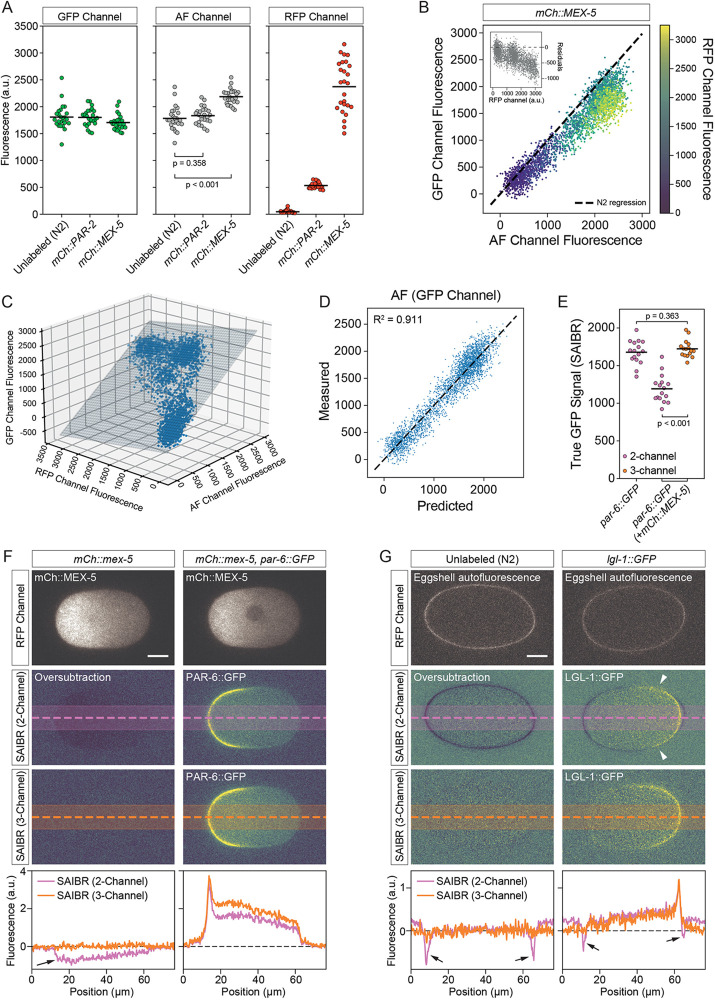
**Spectral AF correction is compatible with dual color labeled samples.** (A) Red fluorophore spillover increases signal in the AF channel for highly expressed proteins. GFP, AF and RFP channel signal shown for TH209 (mCh::PAR-2) or NWG0033 (mCh::MEX-5) embryos relative to unlabeled N2. The increase in the AF channel is significant for NWG0033 due to higher levels of mCherry expression. *P* values were determined using an unpaired two-tailed *t*-test. (B) mCherry spillover shifts the AF versus GFP correlation. Measured signal in Gaussian-filtered (radius=1) AF versus GFP channel for a single NWG0033 (mCh::MEX-5) embryo compared with wild-type N2 control, color-coded by RFP fluorescence (mCherry). Pixels from a region of interest comprising the entire embryo and a small section of surrounding background were specified, and a random selection comprising 10% of all pixels is shown. Dashed line indicates AF versus GFP channel correlation obtained from unlabeled N2 embryos. Inset shows residuals as a function of mCherry expression. (C) Multiple regression fit for three-channel SAIBR of AF signal in the GFP channel as a combined function of signal in the AF and RFP channels for an embryo expressing only mCh::MEX-5. The same 10% sample of pixels is shown. (D) Predicted versus measured AF signal in the GFP channel based on the fit in C*.* (E) Three-channel SAIBR correction compensates for red fluorophore expression. Comparison of total PAR-6::GFP signal in embryos co-expressing mCh::MEX5 (strain is NWG0119) subject to either two- or three-channel SAIBR compared with two-channel SAIBR applied to embryos expressing PAR-6::GFP alone. Overcorrection due to mCherry excitation by 488 nm illumination conditions results in underestimation of true GFP signal when using two-channel SAIBR. *P* values were determined using an unpaired two-tailed *t*-test. (F) Images/profiles showing elimination of AF overcorrection in MEX-5::mCherry-expressing embryos. Two-channel SAIBR using unlabeled N2 embryos for calibration results in oversubtraction that is visible as negative values (arrow) of NWG0033 (mCherry::MEX-5) and in reduced cytoplasmic signal in NWG0119 (mCh::MEX-5, PAR-6::GFP). Oversubtraction is eliminated with three-channel SAIBR using NWG0033 (mCh::MEX-5) for calibration. (G) Simultaneous correction for embryo AF and egg-shell fluorescence. Eggshell fluorescence is strongly visible in the red fluorescence channel. Use of only two-channel SAIBR (calibration using only GFP and AF channels) results in oversubtraction of the AF signal, visible as negative values in two-channel SAIBR images (arrows). This is eliminated by three-channel SAIBR that takes into account the RFP channel, improving signal of the LGL-1 posterior domain (strain: NWG0285). Arrowheads highlight regions of suppressed LGL-1 signal at the domain boundary due to eggshell-induced AF oversubtraction in two-channel SAIBR that does not occur in three-channel SAIBR. Scale bars: 10 µm

Because SAIBR requires measurement of AF in a red-shifted emission channel, the use of dual fluorophore pairs, such as GFP/mNG together with RFP/mCherry/mKate, can introduce complications. Specifically, because red FPs (RFPs) are weakly excited by typical wavelengths used for GFP excitation, they will contribute to apparent AF, which is captured in the AF channel. As long as this contribution is low, i.e. for weak-to-moderate expression levels, it can be safely ignored (e.g. TH209, PAR-2::mCherry, Fig. 5A). However, at higher expression levels, the contribution of RFP signal to the AF channel becomes significant (e.g. NWG0033, mCherry::MEX-5, Fig. 5A). At such levels, this bleedthrough signal induced a deviation in the mapping of AF between the AF and GFP channels compared with N2 in proportion to the degree of RFP expression (Fig. 5B) and leads to overcorrection for AF in the GFP channel if not properly accounted for (e.g. Fig. 5F, left, two-channel SAIBR). In principle, this bleedthrough signal of RFP in the AF channel is proportional to the concentration of the RFP fusion and therefore is proportional to RFP signal captured using standard RFP illumination (RFP channel, ex^561^/em^630/75^). We can therefore compensate for bleedthrough by using inputs from both the AF channel and the RFP channel to establish a revised three-channel SAIBR correction function (primary, GFP; predictor 1, AF; predictor 2, RFP). To this end, we captured images of embryos expressing only RFP (no GFP) in the GFP, AF and RFP channels, and performed a multiple linear regression in which AF in the GFP channel is a function of signal in both the AF and RFP channels (Figs 2, 5C,D). Applying three-channel SAIBR to embryos expressing PAR-6::GFP with MEX-5::mCherry eliminates oversubtraction and yielded measurements that were nearly identical to those for embryos expressing PAR-6::GFP alone with no increase in data scatter, validating the expanded use of SAIBR to dual-labeled samples (Fig. 5E,F). As a further test of its use in dual-labeled embryos, we used three-Channel SAIBR to follow the relative localization of endogenously tagged LGL-1::GFP and PAR-6::mCherry in time-lapse recordings of early embryos, which previously relied on multi-copy overexpression of LGL-1::GFP (Beatty et al., 2010; Hoege et al., 2010) (Movie 1).

Eggshell fluorescence is another issue in *C. elegans* embryos that sometimes arises and can potentially complicate SAIBR. Eggshell fluorescence is usually relatively minor in the GFP and AF channels, and can often be ignored. However, eggshell fluorescence is variable and may occasionally be significant for some methods of sample preparation and/or mounting (see Materials and Methods). Eggshell fluorescence has a distinct spectral profile compared with the AF we have discussed so far. Indeed, in many respects it behaves similarly to an RFP, although with a broader emission spectrum and hence a stronger bleedthrough into the AF channel. Pronounced eggshell fluorescence is particularly problematic when quantifying fluorophore signal at the plasma membrane because eggshell fluorescence will result in oversubtraction of AF in regions where the membrane and eggshell are in contact (Fig. 5G). However, similar to an RFP, it could be compensated for by treating it as a red fluorophore and applying three-channel SAIBR (Fig. 5G). It is important to note that because the emission spectrum of the eggshell is distinct from RFPs, we could not simultaneously correct for both eggshell and RFP in the same samples.

### SAIBR in late *C. elegans* embryos and larvae

**Fig. 6.**
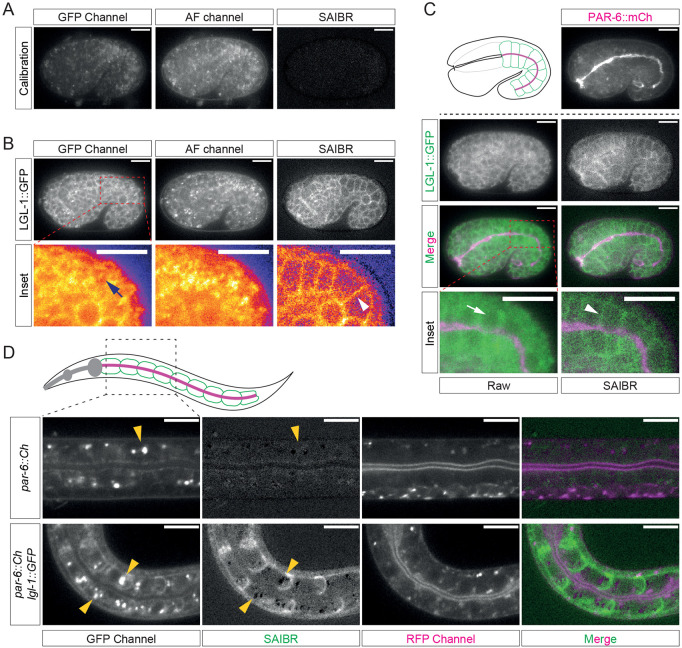
**SAIBR suppresses autofluorescence background in late embryo and larval stages.** (A-C) SAIBR reduces background in late stage *C. elegans* embryos. (A) Lateral view of a 1.5-fold embryo (calibration sample, strain BOX241) highlighting presence of substantial AF, particularly in the posterior near the nascent intestine, which is suppressed by SAIBR. (B) Posterior AF obscures membrane localization of LGL-1::GFP, which is revealed by SAIBR. Images on the bottom row show magnified views of the posterior region (LUT – fire). (C) SAIBR applied to a dual-labeled LGL-1::GFP, PAR-6::mCherry embryo reveals basolateral membrane localization of LGL-1. (Top, left) Schematic of PAR-6 (magenta) and LGL-1 (green) localization in the developing intestine of a 1.5-fold stage embryo. (Top, right) PAR-6::mCherry labeling the apical domain of the intestine. (Second and third rows) Paired uncorrected and SAIBR images shown alone or merged with PAR-6::mCherry visualized in the RFP channel. Images on the bottom row show magnified views of the posterior intestine. (B,C) Different planes of a single 1.5-fold stage embryo (strain NWG0290). In B,C, arrows highlight nuclear void volume surrounded by both fluorophore-specific and autofluorescence signals; arrowheads highlight resolved LGL-1 membrane signal after applying SAIBR. (D) AF correction of *C. elegans* L1 larva reveals LGL-1::GFP basolateral localization in the intestine. Still images of larval intestine expressing either PAR-6::mCherry alone (calibration sample, top row; strain BOX241) or LGL-1::GFP and PAR-6::mCherry (bottom row, NWG0290) shown for RFP (mCherry), GFP and SAIBR-corrected channels along with a merge of RFP/SAIBR channels. Arrowheads indicate gut granules in the GFP and SAIBR channels to highlight their overcorrection in the SAIBR channel. Scale bars: 10 µm.

To expand the applicability of this method, we extended our analysis to late embryos (post-gastrulation) and larval stages. AF is known to be increasingly problematic in intestinal cells as *C. elegans* development proceeds and autofluorescent gut granules are formed (Laufer et al., 1980). In unlabeled 1.5-fold stage embryos, the cells of the embryo posterior were visibly brighter due to AF signal in this region (Fig. 6A). This signal was largely eliminated by SAIBR (Fig. 6A). We next examined the localization of LGL-1::GFP (Fig. 6B,C). Some membrane staining of LGL was visible on the plasma membrane in uncorrected images but was often obscured by AF. Thus, the locations of cells in many areas were only visible due to the reduced AF signal in the nucleus, particularly in the developing intestine (arrows). With SAIBR, the membrane localization of LGL-1::GFP was much more clearly resolved and the cytoplasmic signal significantly more uniform. Notably, basolateral membrane localization of LGL-1 in intestinal cells could be clearly seen, juxtaposed to apical PAR-6::mCherry signal (arrowheads) as observed previously in overexpression lines (Fig. 6C) (Beatty et al., 2010; Sallee et al., 2021).

In L1 larva, gut granules are particularly prominent in intestinal cells in addition to the more-diffuse AF signal characteristic of earlier embryos. Hence, we were curious about how SAIBR would perform (Fig. 6D). When applying SAIBR, we found that a substantial fraction of granule fluorescence was over-subtracted, indicating that the fluorescence profile of mature gut granules differs from the more-diffuse AF signal in the cytoplasm (Fig. 6D, top). This emphasizes the problems associated with the presence of multiple, independently varying sources of AF. Nonetheless, despite modest over-subtraction of some granules, SAIBR significantly improved visualization of LGL-1::-GFP in these tissues (Fig. 6D, bottom), revealing the expected basolateral pattern of localization in intestinal cells (Castiglioni et al., 2020). Thus, despite some over-subtraction of gut granule signal, our method can still improve visualization of weakly expressed fluorophores in both late embryo and larval stages.

### A GUI-based FIJI SAIBR plug-in enables simple AF correction in diverse systems

Although the calibration and correction steps involved in SAIBR are relatively straightforward, we recognize that the need to implement such a workflow may limit widespread adoption. Therefore, to facilitate its use, we have implemented SAIBR as a simple graphical user interface (GUI)-based Fiji plug-in that allows output of AF-corrected images in a few easy steps (summarized in Fig. 2). A detailed description of the plug-in along with full instructions can be found together with sample datasets at https://github.com/goehringlab/saibr_fiji_plugin.

**Fig. 7.**
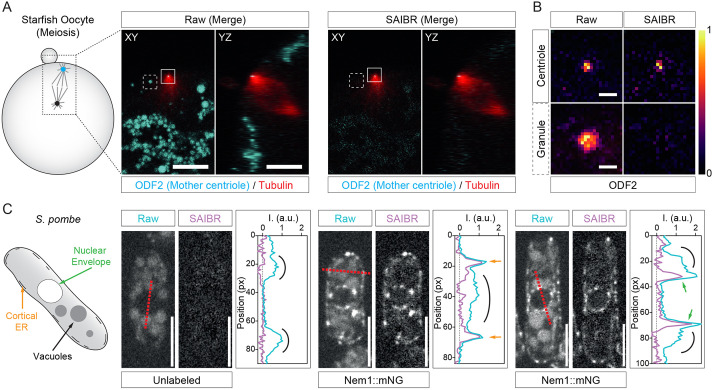
**SAIBR effectively reduces AF in other model systems.** (A,B) Suppression of cortical granule AF in starfish oocytes. (A) Image of a starfish oocyte labeled with ODF2::GFP; rhodamine-tubulin is shown in *xy* and *yz* planes during meiosis II. The spindle (tubulin, red) is perpendicular to the cell cortex with the mother centriole (ODF2, cyan) positioned at the spindle pole near the cell cortex. In raw uncorrected images, yolk granules (cyan) dominate the signal in the GFP channel (left, raw). Yolk granule signal is strongly suppressed by SAIBR, leaving the ODF2-marked centriole as the strongest signal (right, SAIBR). Scale bars: 10 µm. (B) Scaled single channel insets (10× zoom) of the ODF2-marked mother centriole and neighboring yolk granule from A, highlighting specific suppression of yolk granule fluorescence relative to ODF2 (LUT – inferno). Scale bars: 1 µm. (C) Suppression of vacuolar AF in fission yeast cells. Raw (blue) and SAIBR-corrected (purple) midplane images of yeast cells from an unlabeled (left cell) or Nem1::mNG-expressing strain (middle and right cells) imaged in the GFP channel. Quantitation of fluorescence linescans taken across cells as indicated (see red dashed lines). SAIBR reduces non-specific AF signal (mostly emitted from vacuoles – highlighted by black arcs) to background in the unlabeled cell, and reveals a prominent well-resolved Nem1-specific signal at the cortical ER (see orange arrows – middle cell) and nuclear envelope (see green arrows – right cell). Scale bars: 5 µm.

With the SAIBR plug-in in hand, we solicited samples from a variety of experimental systems to validate its general suitability. To this end, we obtained suitable sets of fluorescence images for two systems that exhibit autofluorescence. In starfish oocytes, bright autofluorescent cortical granules dominate the signal in the GFP channel, in this case in comparison with a relatively dim signal for the mother centriole (Fig. 7A). The SAIBR plug-in significantly suppressed the AF signal of granules, typically leaving the centriole clearly visible relative to the residual AF signal (Fig. 7A,B). We observed a similar reduction in AF originating from vacuoles in the fission yeast *S. pombe*, here shown relative to a mNG fusion to the ER/nuclear envelope-localized phosphatase component Nem1 (Fig. 7C). These results confirm the potential broad applicability of SAIBR for AF compensation in cell and developmental systems.

## DISCUSSION

Here, we describe and validate a simple and easy-to-use implementation for autofluorescence correction, SAIBR, provided as a Fiji plug-in. A simplified form of spectral imaging, we leverage the typically broad AF spectrum to allow accurate estimation and correction of AF signal in the GFP channel. SAIBR is platform independent and relies only on commonly available GFP/RFP illumination sources and filter sets. Yet SAIBR yields similar performance to more specialized methods in both single and dual fluorophore-labeled samples, enabling the visualization and accurate quantification of even weakly expressed proteins that are at the limits of detection above AF.

Our aim was to provide a tool that would enable regular and widespread use of AF correction by the research community as part of day-to-day investigation. Autofluorescence is a particularly common problem when imaging GFP in a number of systems with AF sources, including yolk and cortical granules, extracellular matrix, and lysosomal compartments. Although more complex solutions exist, as we have shown, SAIBR provides a simple and straightforward solution that is likely to work efficiently in many contexts. The ease of use of the SAIBR plug-in and the generic imaging conditions required mean that it costs users little to test on their system of choice, allowing one to quickly determine whether a more-complex approach is required.

The simplicity and platform independence of SAIBR allow it to be integrated into a variety of experimental workflows with minimal extra investment of time and resources. We envision that SAIBR and its potential to drive widespread adoption of routine AF correction should enable new experimental approaches. In the *C. elegans* embryo, for example, by allowing accurate AF correction on a per embryo basis, not only will this method provide improved measurement of protein concentration and subcellular distribution within cells, but it will also allow us to address questions related to protein dose, such as assessing variability of protein expression and its potential effects on developmental pathways.

In principle, there is no need to restrict oneself to the wavelengths used here, which were chosen to solve the problem of GFP channel autofluorescence. The technique itself requires only the identification of an appropriate ‘predictor’ channel or channels that can be used to infer AF in the desired fluorophore-reporting ‘primary’ channel, and hence should be well separated from the channel used for fluorophore imaging.

At the same time, simplicity and flexibility come with certain trade-offs. First, SAIBR requires identification of an AF predictor channel that is reasonably well isolated from the primary reporter channel for the fluorophore of interest. Second, it relies on the existence of an AF signal that exhibits a consistent correlation between predictor AF and primary fluorophore channels, and hence is not suited to samples with multiple independently varying sources of AF. Third, SAIBR combines pixel noise from multiple channels, effectively amplifying salt-and-pepper noise in images, although one could, in principle, apply computational denoising strategies to reduce this effect (Krull et al., 2019). Finally, as implemented here, SAIBR requires one to capture images in at least two emission channels, effectively doubling sample illumination and minimum time intervals. The time lag between frames may also lead to pixel mismatches between primary and predictor channels for samples exhibiting rapid motion. However, this last limitation can be bypassed with suitable optics that allow simultaneous capture of multiple emission bands.

There are a variety of approaches to correct for autofluorescence and SAIBR will not and is not intended to replace them, as each has its own advantages and disadvantages. Fluorescence lifetime imaging and spectral unmixing are likely to be substantially better for samples with multiple complex AF signals, but often come with the need for specialized imaging platforms (Zimmermann et al., 2003). Spectral unmixing approaches also require a reference spectrum for each fluorophore to achieve optimal performance. This may not be possible for all samples, although there has been some progress in addressing this problem by ‘blind’ unmixing algorithms (McRae et al., 2019). Another approach is to use feature-based algorithms that seek to define or ‘learn’ characteristics of autofluorescent objects to identify and remove them (Baharlou et al., 2021). However, these are unlikely to deal well with AF, which is not characterized by distinct object features or which spatially overlaps with fluorophore signal, both of which are the case for *C. elegans* embryos. Ultimately, the best choice is likely to be sample dependent, requiring one to quantitatively assess various options as we have here. Minimizing barriers to adoption and testing is key, which is why we have provided our method as a simple, fully open-source and easy-to-use plug-in. In summary, by combining ease of implementation and accurate AF correction with relatively few trade-offs, we hope SAIBR will help facilitate widespread adoption of autofluorescence correction and enable more accurate quantification of the concentration and distribution of fluorescently tagged proteins in cells and tissues.

## MATERIALS AND METHODS

### *C. elegans* – strains and maintenance

*C. elegans* strains were maintained on OP50 bacterial lawns seeded on nematode growth media (NGM) at 20°C under standard laboratory conditions (Stiernagle, 2006). Strains are listed in Table S1. OP50 bacteria were obtained from CGC. Oocytes and zygotes were obtained from hermaphrodites unless otherwise noted. Analysis of zygotes precludes determination of animal sex.

### *C. elegans* – strain construction

Mutation by CRISPR-Cas9 was performed based on the protocol published by Dokshin et al. (2018). Briefly, tracrRNA (IDT DNA, 0.5 µl at 100 µM) and crRNA(s) for the target (IDT DNA, 2.7 µl at 100 µM) with duplex buffer (IDT DNA, 2.8 µl) were annealed together (5 min, 95°C) and then stored at room temperature until required. PCR products containing the insert DNA sequence (GFP in this instance) and an insert with an additional 130 bp homology to the insertion site were generated, column purified (Qiagen, QIAquick PCR purification kit), mixed in equimolar amounts, denatured by heating to 95°C and annealed thorough slow cooling to room temperature to generate a pool of products with long single-stranded DNA overhangs that act as the repair template. An injection mix containing Cas9 (IDT DNA, 0.5 µl at 10 mg/ml), annealed crRNA, tracrRNA and the repair template was incubated at 37°C for 15 min and centrifuged to remove debris (15 min, 14,100 ***g***). Young gravid N2 adults were injected along with a *dpy-10* co-CRISPR injection marker (Arribere et al., 2014) and mutants identified by PCR and sequence verified. Resulting lines were backcrossed with N2s twice before use. Sequences are available in Table S1.

### *C. elegans* – dissection and mounting for microscopy

For most experiments, early embryos were dissected from gravid hermaphrodites in 5-6 µl of M9 buffer (22 mM KH_2_PO_4_, 42 mM NaHPO_4_, 86 mM NaCl and 1 mM MgSO_4_) on a coverslip and mounted under 2% M9 agarose pads (Zipperlen et al., 2001). In some instances (Figs 1B,G, 3A,B,E and 4, and Fig. S1), to minimize eggshell autofluorescence that may be prominent with agarose mounts, embryos were dissected in 8-10 µl of egg buffer [118 mM NaCl, 48 mM KCl, 2 mM CaCl_2_ 2 mM MgCl_2_ and 25 mM HEPES (pH 7.3)], and mounted with 20 µm polystyrene beads (Polysciences) between a slide and coverslip as described previously (Rodriguez et al., 2017).

To harvest late embryos (Fig. 6A-C), gravid worms were allowed to lay embryos for 4-5 h at 20°C. Embryos were collected and mounted in 8-10 µl of egg buffer supplemented with 18.8 µm polystyrene beads (Polysciences).

L1 larva (Fig. 6D) were collected from plates where gravid adult worms were allowed to lay eggs for 12-13 h at 20°C. Whole larva were then mounted between a 2% M9 agarose pad and coverslip in M9 containing 0.1 µm polystyrene beads (Polysciences) and 10 mM levamisole to induce worm paralysis (Reich et al., 2019).

### *C. elegans* – fluorescence microscopy

Unless specified otherwise, midsection confocal images were captured on a Nikon TiE with a 60×/1.40 NA oil objective, further equipped with a custom X-Light V1 spinning disk system (CrestOptics) with 50 µm slits, Obis 488/561 fiber-coupled diode lasers (Coherent) and an Evolve Delta EMCCD camera (Photometrics). Imaging systems were run using Metamorph (Molecular Devices) and configured by Cairn Research. Filter sets were from Chroma: ZT488/561rpc, ZET405/488/561/640X, ET535/50m and ET630/75m. For late embryo and larval imaging, 1.5× magnification was applied (using TiE intermediate magnification switching).

Images were typically captured sequentially, although GFP and AF channels could alternatively be captured simultaneously with a suitable optical setup to minimize the effects of sample movement and bleaching.

Midsection wide-field fluorescence images were captured on a Nikon TiE with a 60×/1.40 NA oil objective, further equipped with a Spectra-X Light Engine (Lumencor). Imaging systems were run using Metamorph (Molecular Devices) and configured by Cairn Research. Filter sets were from Chroma: ET490/20x, ET525/50m and ET632/60m.

To obtain the emission spectra shown in Fig. 1C, embryos were imaged under 488 nm excitation at consecutive 20 nm wavebands over a range of 510-710 nm (yielding lambda stacks of 10 images per embryo). Wavelength-scans were performed on a Leica TCS SP8 inverted microscope equipped with an Apo CS2 63x/1.40 NA oil objective and a HyD detection system. Imaging was managed with LAS X software (Leica Microsystems), and acquisition was set at a scanning speed of 700 Hz with pinhole aperture set to 2 AU.

For midsection confocal imaging of mNG-expressing embryos and comparison between 488 nm and 514 nm excitation configurations (see Fig. 4), experiments were performed on a Leica SP8 microscope (as above) at the indicated emission filter settings. Acquisition was set at a scanning speed of 600 Hz and pinhole aperture was set to 3 AU.

For linear unmixing and emission band optimization, we captured midplane images on an inverted Zeiss LSM880, equipped with Plan-Apochromat 63×/1.4 Oil DIC M27 using 1.7× zoom (0.155μm/ pixel). Imaging was managed with Zen Black software, with a pixel dwell time of 4.10 μs (10.07 s scan time), line averaging of 4 and a pinhole size of 1 AU. Samples were excited at 488 nm and emission captured for ∼9 nm wavebands spanning ∼410-695 nm yielding 32 images. For calibrated unmixing, unlabeled N2 embryos and myo-2::GFP in the pharynx of adult animals were used to define the reference AF and GFP spectra, respectively. Comparative datasets for SAIBR analysis were obtained by summing intensities for the relevant wavebands (broadband: GFP, em^499-562^; AF, em^579-668^; narrowband: GFP, em^499-508^; AF, em^588-597^).

### *C. elegans* – image processing and quantification

In some cases (Fig. 1A-F, Fig. 3, Fig. 5, Figs S2 and S3), images of embryos/samples were taken alongside a local background image (with no samples in the field of view), which was subtracted from the image before analysis. This step can usually be omitted without much detriment, as an even and consistent background signal can be factored into the calibration parameters. However, background subtraction may improve images in cases where the background signal is uneven or variable. In some cases, a median filter (diameter 1-2 px) was applied before incorporation in figures.

Whole-embryo fluorescence intensities are defined as the mean pixel intensity within a manually defined region of interest (ROI) encompassing the embryo. Individual cross-membrane profiles were extracted by taking 50-pixel line profiles perpendicular to and centered on the plasma membrane, using bicubic spline interpolation. Profiles were taken at pixel-width intervals around the circumference of the embryo, and averaged over the posterior-most (Fig. 3E) or anterior-most (Fig. 3F,G) 30% of the circumference of the embryo.

### SAIBR – autofluorescence correction

In the following discussion, the GFP channel is the primary channel for which we want to apply AF correction, and the AF and RFP channels are predictor channels, which we use to predict autofluorescence in the primary channel. In principle, one can adapt the method to any suitable set of primary plus predictor channels.

#### Two channels

Fluorescence signal in the GFP Channel (*G_Observed_*) of an image can be described as a linear sum of true GFP (*G_GFP_*) and autofluorescence (G_AF_) contributions:

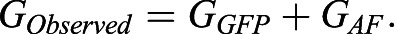
The aim of this procedure is to estimate *G_AF_* in a given image, either on a whole-sample or on a pixel-by-pixel basis, so that this can be subtracted and *G_GFP_* determined. Our method exploits the fact that autofluorescence has a broad emission spectrum, and therefore can be captured in a red-shifted AF channel (A):

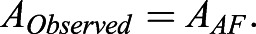


We assume the contribution of GFP to this channel to be very small (*A_GFP_*∼0), owing to the narrow emission spectrum of GFP (however, this assumption can be relaxed, as described in the section ‘GFP spillover correction’). Assuming that autofluorescence can be described as a single component with a characteristic emission spectrum, *G_AF_* and *A_AF_* (=*A_Observed_*) are expected to be linearly proportional:

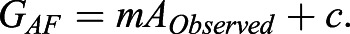
Therefore, provided that the appropriate inter-channel conversion factors (*m* and *c*) are known, *G_AF_* can be calculated from *A_Observed_* using the above equation. Parameters *m* and *c* can be determined by performing linear regression on data from GFP-free samples (for which *G_Observed_*=*G_AF_*), as described in the section ‘Calculation of inter-channel correction factors’. The *c* parameter is included to account for potential differences in background intensity between the two channels.

#### Three channels

The above analysis can break down in samples containing red fluorophore, which can be weakly excited by 488 nm lasers, therefore adding an extra signal component to the A channel (*A_RFP_*):

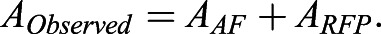
We can account for this contribution by using the RFP channel (*R*), which is specific for RFP fluorophore (*R_GFP_*∼0) and typically free of autofluorescence (*R_AF_*∼0), as an independent readout of RFP levels:

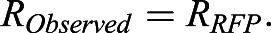
As *A_RFP_* and *R_RFP_* (=*R_Observed_*) are expected to be linearly proportional, *A_AF_*, and therefore *G_AF_* can be described as linear functions of *A_Observed_* and *R_Observed_*:

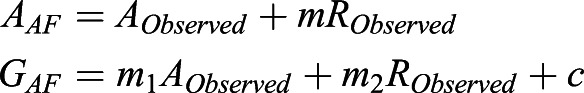
Therefore, provided that the appropriate inter-channel conversion factors (*m*_1_, *m*_2_ and *c*) are known, *G_AF_* can be calculated from *A_Observed_* and *R_Observed_* using the above equation. The determination of these parameters is described below. As before, the *c* parameter is included to account for potential differences in background intensity between the channels.

### Calculation of inter-channel correction factors

For two-channel SAIBR, correction parameters were calculated by performing the following linear regression on data from unlabeled samples (for which *G_Observed_*=*G_AF_*):

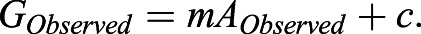
Where a red fluorophore is present, bleedthrough into the A channel must also be accounted for by using a three-channel method. These parameters were obtained by performing multiple linear regression using three-channel data from appropriate RFP-only samples:

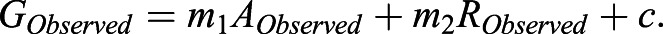
In this study, we used two methods to provide data for the regressions for *C. elegans* embryos: whole-embryo and pixel-by-pixel. In the whole-embryo method, *G_Observed_* and *A_Observed_* (and *R_Observed_*) represent mean whole-embryo intensity measurements for a series of embryos (see *C. elegans* – image processing and quantification). In the pixel-by-pixel method, *G_Observed_* and *A_Observed_* (and *R_Observed_*) represent pixel values taken from manually selected ROIs covering the entire embryo and part of the background, pooled from at least three embryos. We noted that using raw pixel values yielded relatively low correlations between channels, but that correlations increased by first applying a Gaussian filter to the images to reduce salt-and-pepper noise (Fig. S2). A Gaussian radius of between one and two pixels was used for all analysis in this study. Linear regressions were performed using an ordinary least squares method. The plug-in uses the latter pixel-by-pixel method.

Calibration samples were taken contemporaneously with test samples and imaged under identical conditions, as changes to excitation/emission parameters will necessarily alter the parameters of the correction function.

### GFP spillover correction

The long tail of the GFP emission spectrum means that a small fraction of GFP emission will appear in the AF channel. This spillover of GFP signal into the AF channel will artificially inflate *A_AF_* and therefore result in oversubtraction upon application of SAIBR. However, because the magnitude of this effect is always proportional to GFP concentration, it simply rescales the magnitude of *G_GFP_* and thus can be safely ignored for normalized data or for relative comparisons between different GFP-containing samples. Alternatively, the magnitude of this effect can be measured and a correction applied. See Fig. S3 for additional details.

### Additional methods – starfish oocytes

Starfish (*P. miniata*, also known as *A. miniata*) oocyte collection and injection were performed as described previously (Borrego-Pinto et al., 2016a; Terasaki, 1994). Briefly, starfish were obtained from the Southern California Sea Urchin Company (Marinus Scientific, South Coast Bio-Marine) or the Monterey Abalone Company and maintained in seawater tanks at 16°C at the European Molecular Biology Laboratory (EMBL) Marine Facility. The mRNA encoding fluorescent mother centriolar Odf2::mEGFP (https://www.ncbi.nlm.nih.gov/nuccore/1040843242) (Borrego-Pinto et al., 2016b) was injected the day before imaging, while Cy3 tubulin (a gift from the Nédélec laboratory, EMBL, Heidelberg, Germany) was injected shortly before imaging. After meiotic maturation with 10 µM 1-methyladenine (Acros Organics), oocytes were imaged on a Leica SP5 confocal microscope, as described previously (Borrego-Pinto et al., 2016b). Sequential scanning was performed: in a first scan, 488 nm excitation was coupled to both mEGFP (primary) and red-shifted (predictor 1) emission channels to record Odf2-mEGFP and autofluorescence, respectively. In a second scan, 561 nm excitation was combined with the same red-shifted emission channel to record Cy3-tubulin fluorescence (predictor 2).

### Additional methods – *S. pombe*

*Schizosaccharomyces pombe* (*S. pombe*) cells were grown in YES (yeast extract with supplements) medium overnight at 30°C. Prior to imaging, 1 ml *S. pombe* cell culture with OD_595nm_ 0.4-0.6 was concentrated to 50 µl after centrifugation at 1500 ***g*** for 30 s. 2 µl of cell suspension were loaded under a 22×22 mm glass coverslip (VWR, thickness: 1.5). Spinning disk confocal images of *S. pombe* were captured with an Eclipse Ti-E inverted microscope fitted with a Yokogawa CSU-X1 spinning disk confocal scanning unit, 600 series SS 488 nm, SS 561 nm lasers, single band filters FF01-525/50-25 and FF01-617/73-25 (Semrock Brightline), Nikon CFI Plan Apo Lambda 100x (NA=1.45) oil objective and an Andor iXon Ultra U3-888-BV monochrome EMCCD camera. Image acquisition was controlled by Andor IQ3 software.

## Supplementary Material

Supplementary information

Reviewer comments
