# Supplementary material for: SAIBR: a simple, platform-independent method for spectral autofluorescence correction
Source: Development. 2022 Jul 14;149(14):dev200545. doi: 10.1242/dev.200545 (PMC9445497; doi:10.1242/dev.200545)
Supplement: Supplementary information [file develop-149-200545-s1.pdf]

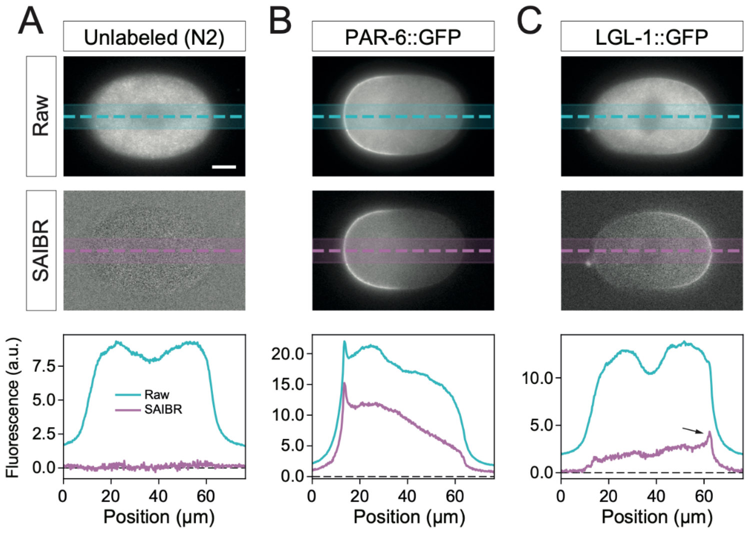

Fig. S1. Spectral AF correction using widefield fluorescence microscopy. Raw (top) and SAIBR-corrected (middle) midplane images of **(A)** unlabeled wild-type (N2) control, **(B)** PAR-6::GFP (strain: KK1248), and **(C)** LGL-1::GFP (strain: NWG0285) zygotes imaged with widefield fluorescence microscopy. (bottom) Quantification of fluorescence linescans taken across the embryos as indicated. Arrow highlights plasma membrane signal in LGL-1::GFP linescan that is practically undetectable in uncorrected images. Scale Bars = 10 μm.

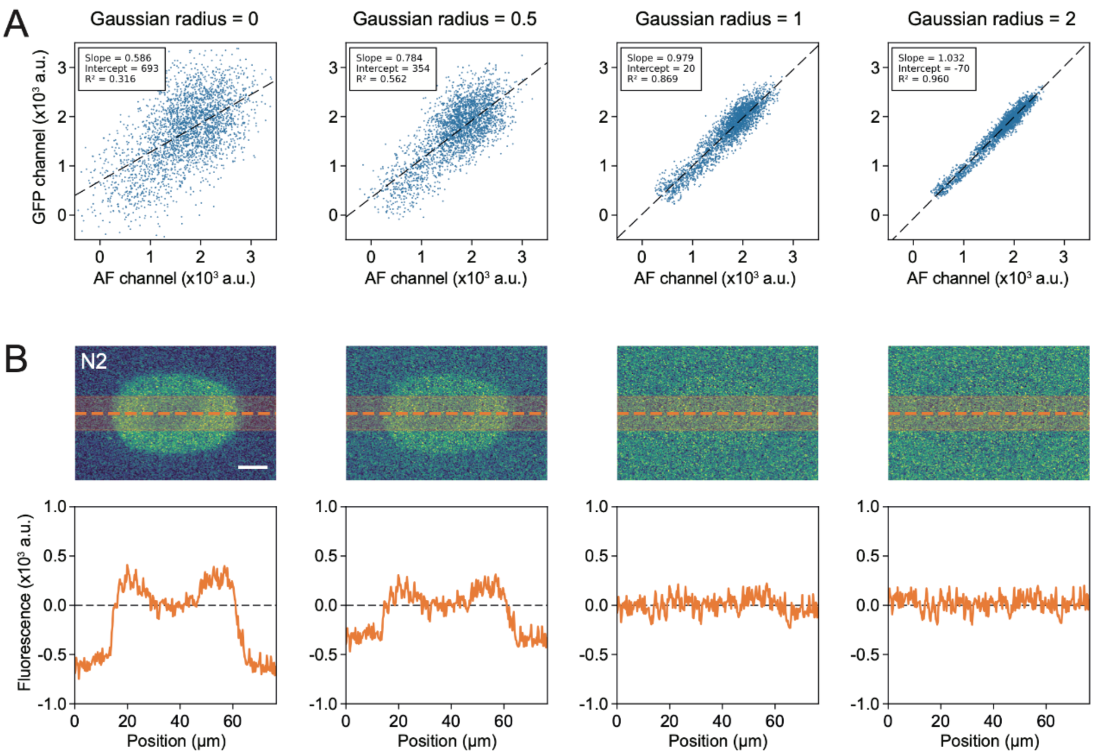

Fig. S2. Suppression of shot noise improves per-pixel AF to GFP Channel correlation. (A) Per-pixel plots of AF vs GFP Channel signal for images of unlabeled embryos subject to gaussian blur of indicated radius. Linear fit shown as dashed line with parameters in inset indicating improved fit with increasing gaussian radius. **(B)** Results on AF correction of unlabeled N2 embryos using the linear fits in (A). Processed images (top) and line scan across the central region of the embryo (bottom) show improved suppression of AF with increasing Gaussian radius.

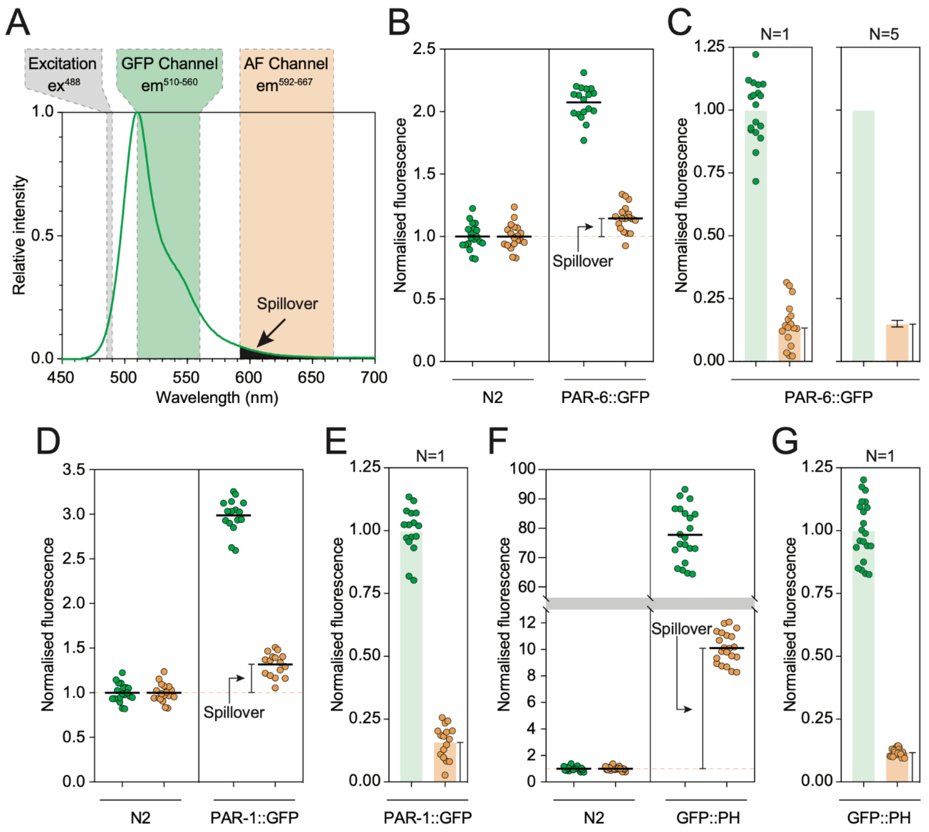

Fig. S3. Quantification of GFP spillover into AF Channel. (A) The long tail of the GFP emission spectrum (data from searchlight.semrock.com) means that some GFP signal will appear in the AF Channel (em$^{592\text{-}667}$). Predicted spillover in the AF Channel shaded in black. **(B-G)** Quantification of the magnitude of GFP spillover into the AF Channel reveals it is a consistent fraction of GFP signal (here ~15%). Spillover was detected by comparing AF Channel signal from unlabeled and GFP-expressing embryos (GFP Channel - green, AF Channel - orange). Mean fluorescence signal for the indicated strains/channels (B, D, E) and spillover as a fraction of normalized GFP signal (C, E, G) shown. Note for PAR-6::GFP (Strain: KK1248), we replicated this measure five times and show both a representative replicate and mean±SD for all five replicates. A single replicate was performed each for PAR-1::GFP (Strain: KK1262) and GFP::PH (Strain: LP306) for comparison. While this spillover of GFP signal into the AF Channel will result in oversubtraction when applying SAIBR, because the magnitude of this effect is always proportional to GFP concentration, it simply rescales the magnitude of the obtained GFP value and thus is irrelevant for normalized data or for comparisons between different GFP-containing samples.

Development • Supplementary information

Table S1. Strains and Reagents

**Oligonucleotides**	
**Description**	**Sequence**
lgl-1 crRNA #1	CTCCGAACTACAGCGAAGTA
lgl-1 crRNA #2	ACGGTGAATTTGAACTTTCG
GFP fwd ODN	ATGAGTAAAGGAGAAGAATTGTT
GFP rev ODN	CTTGTAGAGCTCGTCCATTCC
lgl-1 HDR fwd ODN	TCTTCATTTGAGTCGAGGAAGTTTATAGACCCAATCGACGTAATGGCACTAAACTCGAGTTGTTTAT CACGTGATGGTGACGTGGCATTCCTCATCAAATCCTCCGAACTACAGCGAAGTACAGTAAACCTCA ATTTCAGAGGTTGGAGCAGTACGCACAAGTCAATGAGTAAAGGAGAAGAATTGTT
lgl-1 HDR rev ODN	CAAAAAAGGCAAAGACCGAGGGCAAATAAATAACATAATAAAGTTTAAAAAAAACCACCATTTCAAA CAAAATTAATATATATCAACAGGAAAACGATTTTTAAAAAAAATGCATCTACTTGTAGAGCTCGTCCAT TCCG

***C. elegans* strains**		
**Strain**	**Genotype**	**Source**
N2	Wild type	Caenorhabditis Genetics Center (CGC)
BOX241	*par-6(mib25[par-6::mcherry-LoxP]) I*	Mike Boxem<br>(Castiglioni et al., 2020; Reich et al., 2019)
DG4190	*cdc-25.3(tn1712[gfp::3xflag::cdc-25.3]) III*	CGC/David Greenstein<br>(Tsukamoto et al., 2017)
JH2840	*axIs??? [nmy-2p::pgl-1::GFP::patr-1::nmy-2 3'UTR]. axIs1731 [pie-1p::mCherry::mex-5::pie-1 3'UTR + unc-119(+)].*	CGC/Geraldine Seydoux<br>(Gallo et al., 2010)
KK1216	*par-3(it298[par-3::gfp]) III*	CGC/Ken Kemphues (Rodriguez et al., 2017)
KK1248	*par-6(it310[par-6::gfp]) I*	CGC/A Syed & Ken Kemphues<br>(Rodriguez et al., 2017)
KK1262	*par-1(it324[par-1::gfp::par-1 exon 11a]) V*	CGC/Dianne Morton (Rodriguez et al., 2017)
LP216	*par-6(cp45[par-6::mneongreen::3xFlag + LoxP unc-119(+) LoxP]) I; unc-119(ed3) III*	CGC/Dan Dickinson<br>(Dickinson et al., 2017)
LP306	*cpIs53 [mex-5p::gfp-C1::plc(delta)-PH::tbb-2 3'UTR + unc-119 (+)] II; unc-119(ed3) III*	CGC/Bob Goldstein<br>(Heppert et al., 2016)
NWG0033	*unc-119(ed3) III; axIs1731[pie-1p::mcherry::mex-5::pie-1 3'UTR + unc-119 (+)]*	Derived from JH2840
NWG0119	*par-6(it310[par-6::gfp]) I; unc-119(ed3) III; axIs1731[pie-1p::mcherry::mex-5::pie-1 3'UTR + unc-119 (+)]*	This paper

NWG0285	*lgl-1(crk66[lgl-1::gfp]) X*	This paper
NWG0286	*lgl-1(crk67[lgl-1::gfp]) X*	This paper
NWG0290	*par-6(mib25[par-6::mcherry-LoxP]) I; lgl-1(crk67[lgl-1::gfp]) X*	This paper
TH209	*unc-119(ed3) III; ddIs31[pie-1p::mcherry::par-2; unc-119(+)]*	Hyman Lab (Brangwynne et al., 2009)
***S. pombe* strains**		
**Strain**	**Genotype**	**Source**
SO2865	ade6-210 ura4-D18 leu1-32 h+	Snezhana Oliferenko
SO8304	nem1-mNeonGreen::kanR ade6-216 ura4-D18 leu1-32 h-	Snezhana Oliferenko

Development • Supplementary information

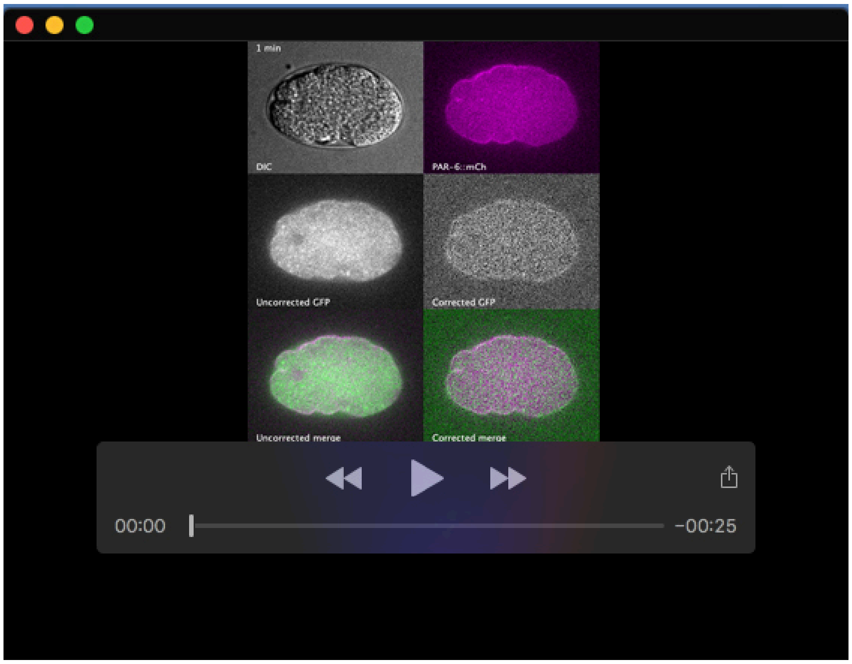

Movie 1. Timelapse of *C. elegans* embryo expressing LGL-1::GFP, PAR-6::mCherry at the endogenous loci from the 1- to 4-cell stage highlighting differences between uncorrected and corrected image stacks for the GFP channel. DIC, mCherry, raw GFP and SAIBR images shown. Frame rate 1/min.

Supplemental References

Brangwynne, C. P., Eckmann, C. R., Courson, D. S., Rybarska, A., Hoege, C., Gharakhani, J., Jülicher, F. and Hyman, A. A. (2009). Germline P granules are liquid droplets that localize by controlled dissolution/condensation. **324**, 1729–1732.

Castiglioni, V. G., Pires, H. R., Rosas Bertolini, R., Riga, A., Kerver, J. and Boxem, M. (2020). Epidermal PAR-6 and PKC-3 are essential for larval development of C. elegans and organize non-centrosomal microtubules. *eLife* **9**, e62067.

Dickinson, D. J., Schwager, F., Pintard, L., Gotta, M. and Goldstein, B. (2017). A Single-Cell Biochemistry Approach Reveals PAR Complex Dynamics during Cell Polarization. *Dev. Cell* **42**, 416-434.e11.

Gallo, C. M., Wang, J. T., Motegi, F. and Seydoux, G. (2010). Cytoplasmic partitioning of P granule components is not required to specify the germline in C. elegans. *Science* **330**, 1685–1689.

Heppert, J. K., Dickinson, D. J., Pani, A. M., Higgins, C. D., Steward, A., Ahringer, J., Kuhn, J. R. and Goldstein, B. (2016). Comparative assessment of fluorescent proteins for in vivo imaging in an animal model system. *Mol. Biol. Cell* **27**, 3385–3394.

Reich, J. D., Hubatsch, L., Illukkumbura, R., Peglion, F., Bland, T., Hirani, N. and Goehring, N. W. (2019). Regulated Activation of the PAR Polarity Network Ensures a Timely and Specific Response to Spatial Cues. *Curr. Biol.* **29**, 1911-1923.e5.

Rodriguez, J., Peglion, F., Martin, J., Hubatsch, L., Reich, J., Hirani, N., Gubieda, A. G., Roffey, J., Fernandes, A. R., St Johnston, D., et al. (2017). aPKC Cycles between Functionally Distinct PAR Protein Assemblies to Drive Cell Polarity. *Dev. Cell* **42**, 400-415.e9.

Tsukamoto, T., Gearhart, M. D., Spike, C. A., Huelgas-Morales, G., Mews, M., Boag, P. R., Beilharz, T. H. and Greenstein, D. (2017). LIN-41 and OMA Ribonucleoprotein Complexes Mediate a Translational Repression-to-Activation Switch Controlling Oocyte Meiotic Maturation and the Oocyte-to-Embryo Transition in Caenorhabditis elegans. *Genetics* **206**, 2007–2039.

Development • Supplementary information
