# Supplementary material for: SAIBR: a simple, platform-independent method for spectral autofluorescence correction
Source: Development. 2022 Jul 14;149(14):dev200545. doi: 10.1242/dev.200545 (PMC9445497; doi:10.1242/dev.200545)
Supplement: Reviewer comments [file develop-149-200545_review_history.pdf]

SAIBR: a simple, platform-independent method for spectral autofluorescence correction

Nelio T. L. Rodrigues, Tom Bland, Joana Borrego-Pinto, KangBo Ng, Nisha Hirani, Ying Gu, Sherman Foo and Nathan W. Goehring
DOI: 10.1242/dev.200545

Editor: Swathi Arur

Review timeline
Original submission: 19 January 2022
Editorial decision: 8 March 2022
First revision received: 12 May 2022
Accepted: 6 June 2022

Original submission

First decision letter

MS ID#: DEVELOP/2022/200545

MS TITLE: SAIBR: A simple, platform-independent method for spectral autofluorescence correction

AUTHORS: Nelio T.L. Rodrigues, Tom Bland, Joana Borrego-Pinto, KangBo Ng, Nisha Hirani, Ying Gu, Sherman Foo, and Nathan W Goehring

I have now received all the referees reports on the above manuscript, and have reached a decision. The overall evaluation is positive and we would like to publish a revised manuscript in Development, provided that the referees' comments can be satisfactorily addressed. Please attend to all of the reviewers' comments in your revised manuscript and detail them in your point-by-point response. If you do not agree with any of their criticisms or suggestions explain clearly why this is so.

Reviewer 1

Advance summary and potential significance to field

The manuscript by Rodrigues and colleagues presents a simple method to reduce autofluorescence signal in imaging data. The method principally aims to reduce autofluorescence in the green (GFP) channel. It relies on acquisition of a second image with the same excitation light but different emission filter sets (here with the filter sets typically used to acquired RFP signal), capturing a significant autofluorescence emission light but only the tail-end of the GFP emission. Through a re-scaling function, obtained by correlating the signal in the autofluorescence and GFP channels (in samples without GFP), this signal can be used to subtract it from the GFP channel on a pixel-per-pixel basis and recover an image where the autofluorescence has been largely removed. The authors also made the method ready-to-use by building a simple FIJI plugin. This will help wide use of the method. The manuscript is by and large convincing and well written. I have only suggestions for relatively minor changes, largely to clarify the limitations of the method.

© 2022. Published by The Company of Biologists under the terms of the Creative Commons Attribution License (https://creativecommons.org/licenses/by/4.0/).

Comments for the author

First, it should be specified early in the text, already in the abstract, that the method permits correction in single fluorescence channels. In the way the abstract is currently formulated, one is led to believe that all autofluorescence will be corrected by the method.
Second, please specify in the description of the method (at the beginning of result section) the need to acquire two consecutive images. The discussion briefly mentions the problem of signal bleaching and movement of autofluorescent structures between acquisitions, but this can be a serious issue. Could the two channels not be acquired simultaneously to both reduce bleaching of the sample and movement issues between frames? In any case, this should be made clear early in the text.
Third, one aspect that is unclear is why signals with different spectra/intensity cannot be simultaneously subtracted. The authors first state this when extending the method to double-labelled samples (GFP and RFP) and show the example of RFP and eggshell. It is not clear to me why the eggshell is not visible in Fig 4F and why RFP and eggshell signals cannot be simultaneously subtracted from the AF channel. Similarly, in the analysis of gut granules in Fig 5D, it is not clear to me whether SAIBR was performed using zygote or gut (containing autofluorescent granules) images from unlabelled sample, and, in the latter case, why over-correction is observed.
Fourth, to help implementation of the method, a limiting factor is the identification of the autofluorescence channel. In the examples shown, the autofluorescence channel is simply the RFP filter sets with GFP excitation, but autofluorescence may not be limited to the GFP channel or may have other spectrum in other species. For instance, chloroplasts show very strong autofluorescence in the red. Guidelines for how to best choose the autofluorescence channel would be helpful.
Finally, to extend the method, it would be valuable to be also able to remove the autofluorescence not only from the GFP channel, but also from the red channel (or any other channel), when performing two-colour imaging. I understand that red channel autofluorescence is less problematic in C. elegans, but it could be significant in other cells and organisms. Again, chlorophyll fluoresces for instance in both green and red channels, and correction of both in the same sample would be useful.

Minor comments
In the figures, arrows and arrowheads are sometimes too small to be easily visible (for instance Fig 5 and 6, where colours of the arrows become impossible to read). In Fig 6, coloured frames around images corresponding to coloured traces are also quasi-invisible. Perhaps better to colour the "raw" and "SAIBR" text.
The acknowledgement weirdly states that “This research was funded in whole, or in part, by the Wellcome Trust”. Surely the authors must know which to state!

<u>Reviewer 2</u>

Advance summary and potential significance to field

Fluorescence imaging is an important technique for observing the localization of specific proteins and monitoring biological processes in cells and tissues.
However, many organisms and cell types exhibit intrinsic autofluorescence (AF) that can obscure localization to specific structures and provides a significant barrier to downstream image analysis. Therefore, there is an unmet need for the development of user-friendly and easily implemented methods for excluding AF from microscopy images. Here Rodrigues et al. describe a new and straightforward protocol to correct for AF using standard filter sets and imaging conditions that enables accurate detection and quantitation of fluorescence signal. The authors leverage the distinct spectral properties of AF to estimate and correct for AF signal in the GFP channel, termed Spectral Autofluorescence Image correction By Regression (SAIBR). Importantly, this approach is platform independent and is provided as a plugin to the commonly used and freely available FIJI software. The authors demonstrate that the SAIBR approach can be used with C. elegans embryos expressing either single or dual fluorescent proteins throughout different stages of development, and is also broadly applicable to imaging datasets from other systems such as starfish oocytes and fission yeast. The authors’ conclusions are supported by the data and the experiments are thoroughly controlled throughout.

© 2022. Published by The Company of Biologists under the terms of the Creative Commons Attribution License (https://creativecommons.org/licenses/by/4.0/).

Together, this is an intriguing study that provides a new avenue to facilitate integration of AF correction as a standard part of imaging workflows. Therefore these findings will be of significant interest to the broader scientific community.

Comments for the author

Below are suggestions for a few points of clarification.
In Box 1 – the authors state that "fSAIBR is defined by imaging multiple unlabeled calibration samples". Is there a recommendation for the number of calibrated samples that should be imaged to achieve optimal results with fSAIBR? Does this vary based on the experiment and specimen? This information would be very helpful to the end user looking to implement SAIBR.
While the authors nicely show that SAIBR provides a significant advancement for estimation and correction of AF, inclusion of additional comparisons as part of the "Benchmarking against alternative strategies section" may be helpful for the reader. For instance, comparison of SAIBR with spectral un-mixing would highlight the increased performance of SAIBR without the need for more specialized equipment.
Likewise, comparison of SAIBR to other AF correction strategies, such as Baharlou et al. 2020 (Autofluorescence Identifier) that aims to remove specific AF objects from microscopy images and is also implemented using ImageJ/Fiji, may highlight the superior performance of SAIBR to account for intra- and inter-embryo AF variation.
Can the authors comment if SAIBR can also be applied to correct for AF in time-lapse imaging datasets?

Minor:
For the PAR-6, PAR-3, LGL-1 lines described in the first paragraph (pg4, lines 124-129) of the results section, it is not clear to the reader if these C. elegans lines were generated in a previous study or created during the course of this work. If not generated in this study, the authors should add references for these lines.
On pg9, GUI should be defined, as it is the first time this term is used in the manuscript.
In Fig 6 panel C, the arrows highlighting specific regions on the fluorescence linescans for the raw and SAIBR corrected samples are currently very difficult to see (especially for Nem1::mNG). The authors should consider alternatives ways to denote these regions.

First revision

Author response to reviewers' comments

Reviewer Comments:

We would like to thank both reviewers for taking the time to provide careful and constructive comments on our manuscript. To address these points, we have improved clarity, added additional instructions for the method, and performed additional analysis (Figure 3), which we feel has led to improved readability of the manuscript and enhanced the user friendliness of the method. Detailed response can be found below.

Reviewer 1:

The manuscript by Rodrigues and colleagues presents a simple method to reduce autofluorescence signal in imaging data. The method principally aims to reduce autofluorescence in the green (GFP) channel. It relies on acquisition of a second image with the same excitation light but different emission filter sets (here with the filter sets typically used to acquire RFP signal), capturing a significant autofluorescence emission light but only the tail-end of the GFP emission. Through a re-scaling function, obtained by correlating the signal in the autofluorescence and GFP channels (in samples without GFP), this signal can be used to subtract it from the GFP channel on a pixel-per-pixel basis and recover an image where the autofluorescence has been largely removed. The authors also made the method ready-to-use by building a simple FIJI plugin. This will help wide use of the method. The manuscript is by and large convincing and well written. I have only suggestions

© 2022. Published by The Company of Biologists under the terms of the Creative Commons Attribution License (https://creativecommons.org/licenses/by/4.0/).

for relatively minor changes, largely to clarify the limitations of the method.

Reviewer 1 Comments for the Author:
First, it should be specified early in the text, already in the abstract, that the method permits correction in single fluorescence channels. In the way the abstract is currently formulated, one is led to believe that all autofluorescence will be corrected by the method.

>> We have modified the abstract to clarify this point.

Second, please specify in the description of the method (at the beginning of result section) the need to acquire two consecutive images. The discussion briefly mentions the problem of signal bleaching and movement of autofluorescent structures between acquisitions, but this can be a serious issue. Could the two channels not be acquired simultaneously to both reduce bleaching of the sample and movement issues between frames? In any case, this should be made clear early in the text.

>> We explicitly addressed precisely this suggestion in the Discussion (see below) and note the need for two channels, both in the text and in Box 1. We have added additional text to the imaging section of the methods to make this even more explicit.

"Finally, as implemented here, SAIBR requires one to capture images in at least two emission channels, effectively doubling sample illumination and minimum time intervals The time lag between frames may also lead to pixel mismatches between GFP and AF channels for samples exhibiting rapid motion. ***However, this last limitation can be bypassed with suitable optics to allow for simultaneous capture of multiple emission bands****."*

Third, one aspect that is unclear is why signals with different spectra/intensity cannot be simultaneously subtracted. The authors first state this when extending the method to double-labeled samples (GFP and RFP) and show the example of RFP and eggshell. It is not clear to me why the eggshell is not visible in Fig 4F and why RFP and eggshell signals cannot be simultaneously subtracted from the AF channel. Similarly, in the analysis of gut granules in Fig 5D, it is not clear to me whether SAIBR was performed using zygote or gut (containing autofluorescent granules) images from unlabelled sample, and, in the latter case, why over-correction is observed.

>> The method explicitly requires one to establish a correlation between AF signal in the GFP and AF channels. If there are multiple sources of AF, one needs a way to separate the contributions of these two signals as they will likely vary independently and exhibit distinct correlation functions. What this means is one typically needs to capture images in as many channels as one has independently varying sources of fluorescence. If the source of AF is different in the GFP and RFP channels, this would require four channels (GFP, GFP AF, RFP, RFP AF). This is a fundamental limit to the SAIBR approach which we address in the Discussion.

Regarding the specific case of the eggshell, RFP and eggshell have different spectral characteristics and vary independently and therefore cannot be subtracted together. One could address this by introducing a fourth channel to quantify eggshell, but this was not possible in our system as eggshell was not detectable in far red and UV/BFP illumination introduced additional AF signals. Eggshell fluorescence is highly variable between samples and we don't understand precisely why. However, in the specific case of Figure 4F, MEX-5 signal is so strong that it tends to mask any eggshell contribution. In practice, it is often best to try to eliminate eggshell fluorescence as we note in the methods.

Regarding granules, we performed calibration with unlabeled images of similar samples (e.g. gut w/ granules present). The main problem is that granules have distinct spectral properties and thus in the gut, we have at least two AF sources: background AF and granules, which are not easily separable. In fact, granules can even vary in their individual spectral signatures. However, granules tend to make up only a minority of the pixels and thus SAIBR calibration favors correction of the background cytoplasmic AF rather than granules (hence the over-subtraction).
One can gate SAIBR calibration on an ROI for a given granule. This allows one to specifically suppress granule AF, but at the expense of worse correction elsewhere in the image. See below:

© 2022. Published by The Company of Biologists under the terms of the Creative Commons Attribution License (https://creativecommons.org/licenses/by/4.0/).

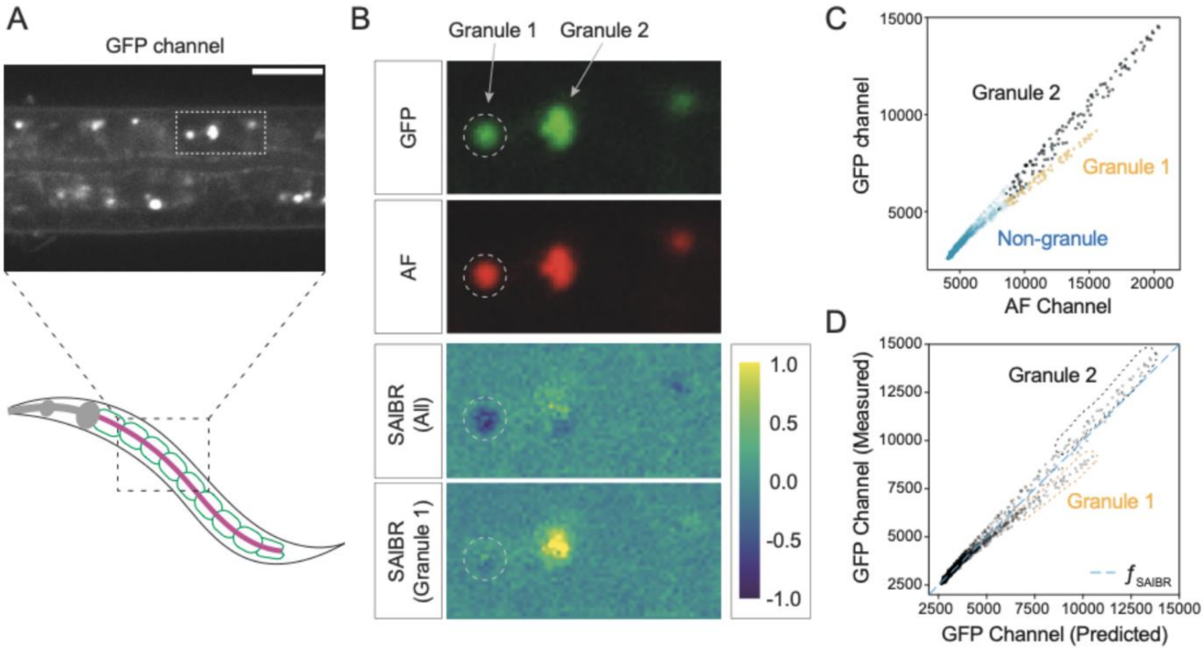

Rebuttal Figure. Granules exhibit distinct and variable correlation between GFP and AF channels leading to over- / under-correction. (A) Schematic of region of interest (ROI) in the L1 intestine. (B) Top: Raw GFP and AF channels highlight two autofluorescent gut granules. Bottom: SAIBR corrected images. Note granule 1 is over-subtracted when SAIBR is calibrated with all pixels (SAIBR All). Calibrating SAIBR using only Granule 1 pixels improves correction of Granule 1, but now Granule 2 is strongly under-subtracted. (C) Plots of pixel intensity in AF and GFP channels shows distinct relationships of Granule 1 and 2 relative to other pixels. (D) This deviation means that when calibrated against all pixels, which will be dominated by cytoplasmic AF, predicted vs. measured values for granule signal deviate from the SAIBR model (dashed blue line).

Fourth, to help implementation of the method, a limiting factor is the identification of the autofluorescence channel. In the examples shown, the autofluorescence channel is simply the RFP filter sets with GFP excitation, but autofluorescence may not be limited to the GFP channel or may have other spectrum in other species. For instance, chloroplasts show very strong autofluorescence in the red. Guidelines for how to best choose the autofluorescence channel would be helpful.

>> We have added a section to the methods (and to the plugin instructions) that provide additional guidance in setting up SAIBR for a given sample (wavelength selection, number of calibration images, etc.).

Finally, to extend the method, it would be valuable to be also able to remove the autofluorescence not only from the GFP channel, but also from the red channel (or any other channel), when performing two-colour imaging. I understand that red channel autofluorescence is less problematic in C. elegans, but it could be significant in other cells and organisms. Again, chlorophyll fluoresces for instance in both green and red channels, and correction of both in the same sample would be useful.

>> As we note in the discussion, the method is agnostic to the channels one can use. One does however need to identify a suitable set of channels for analysis. As we note, the number of channels is typically equal to the number of independent fluorescence signals. If chlorophyll were the only source of AF and could be imaged in a channel distinct from both GFP and RFP, SAIBR should work. However, without testing plant samples, it is difficult to comment on this specific case. Our hope is that as people try the method, that they will let us know what worked / what didn't and we can include these examples in updated documentation. Note we now include a section in the Plugin documentation to list tips/tricks for various samples and we plan to update this regularly.

© 2022. Published by The Company of Biologists under the terms of the Creative Commons Attribution License (https://creativecommons.org/licenses/by/4.0/).

In response to this comment, we realized that the naming of the SAIBR channels as GFP, AF, and RFP (which is how we use them) may give the impression that these are the channels you must use. We have renamed these in the plugin as "Primary", which is the channel of interest from which you want to subtract AF, and "Predictor 1", and "Predictor 2" which are the channels used to accurately predict AF in the Primary channel.

Minor comments

In the figures, arrows and arrowheads are sometimes too small to be easily visible (for instance Fig 5 and 6, where colours of the arrows become impossible to read). In Fig 6, coloured frames around images corresponding to coloured traces are also quasi-invisible. Perhaps better to colour the "raw" and "SAIBR" text.

>> The labeling in Figures 5 and 6 has been updated as suggested.

The acknowledgement weirdly states that "This research was funded in whole, or in part, by the Wellcome Trust". Surely the authors must know which to state!

>> This is the standard funding statement mandated by the funding agency.

Reviewer 2:

Fluorescence imaging is an important technique for observing the localization of specific proteins and monitoring biological processes in cells and tissues.
However, many organisms and cell types exhibit intrinsic autofluorescence (AF) that can obscure localization to specific structures and provides a significant barrier to downstream image analysis. Therefore, there is an unmet need for the development of user-friendly and easily implemented methods for excluding AF from microscopy images. Here Rodrigues et al. describe a new and straightforward protocol to correct for AF using standard filter sets and imaging conditions that enables accurate detection and quantitation of fluorescence signal. The authors leverage the distinct spectral properties of AF to estimate and correct for AF signal in the GFP channel, termed Spectral Autofluorescence Image correction By Regression (SAIBR). Importantly, this approach is platform independent and is provided as a plugin to the commonly used and freely available FIJI software. The authors demonstrate that the SAIBR approach can be used with C. elegans embryos expressing either single or dual fluorescent proteins throughout different stages of development, and is also broadly applicable to imaging datasets from other systems such as starfish oocytes and fission yeast. The authors' conclusions are supported by the data and the experiments are thoroughly controlled throughout. Together, this is an intriguing study that provides a new avenue to facilitate integration of AF correction as a standard part of imaging workflows. Therefore, these findings will be of significant interest to the broader scientific community.

Reviewer 2 Comments for the Author:
Below are suggestions for a few points of clarification.

In Box 1 - the authors state that "fSAIBR is defined by imaging multiple unlabeled calibration samples". Is there a recommendation for the number of calibrated samples that should be imaged to achieve optimal results with fSAIBR? Does this vary based on the experiment and specimen? This information would be very helpful to the end user looking to implement SAIBR.

Overall, we have found that AF correction can work satisfactorily with a single calibration image. However, in practice we usually use 3-5 as this ensures that we can confirm that the relationships are consistent across samples. If these nicely overlay, it also provides a reasonably good sense of how well SAIBR will work. There are a few cases in which we have obtained calibration curves that are highly variable or there is no clear correlation, and these samples were generally not suitable for SAIBR. We now included a section on SAIBR optimization in the Plugin documentation.

While the authors nicely show that SAIBR provides a significant advancement for estimation and correction of AF, inclusion of additional comparisons as part of the "Benchmarking against alternative strategies section" may be helpful for the reader. For instance, comparison of SAIBR

© 2022. Published by The Company of Biologists under the terms of the Creative Commons Attribution License (https://creativecommons.org/licenses/by/4.0/).

with spectral un-mixing would highlight the increased performance of SAIBR without the need for more specialized equipment.

>> We now include additional comparisons to both optimized narrow emission bands and linear unmixing (Figure 3). Overall, SAIBR compares very favorably to linear unmixing, generally performing much better than simply optimizing the emission band or blind demixing and slightly better than reference-calibrated unmixing for our samples, but there are likely instances where the reverse will be true..

Likewise, comparison of SAIBR to other AF correction strategies, such as Baharlou et al. 2020 (Autofluorescence Identifier) that aims to remove specific AF objects from microscopy images and is also implemented using ImageJ/Fiji, may highlight the superior performance of SAIBR to account for intra- and inter-embryo AF Variation.

>> This is a good point. We have added some additional discussion of alternative methods to the discussion to put SAIBR in context and highlight pros and cons. We did try several of these plugins on our images (worm, starfish) in the hopes that we could generate a head-to-head comparison figure, but unfortunately, out of the box, we were unable to get sufficiently satisfactory results from either AFID or LUMOS to enable a reasonable comparison. Given that this could be due to insufficient optimization or specific issues with our sample images, we felt it better to highlight other methods that exist and people can try them out for themselves.

Can the authors comment if SAIBR can also be applied to correct for AF in time- lapse imaging datasets?

>> Yes, in principle there is no restriction on using SAIBR on time-series data assuming that the correction function does not vary in time (i.e. that the AF behaves similarly across timepoints). Note we included just such an example in Movie S1. Specifically, as currently configured, during calibration, the plugin reads in the selected frame. However, once the calibration is specified, if a stack is selected during correction, all slices will be processed, resulting in a corrected time-series dataset. We realize it was not clear in practice how best to implement this and have added comments to methods and GitHub about processing image stacks.

Minor:

For the PAR-6, PAR-3, LGL-1 lines described in the first paragraph (pg4, lines 124-129) of the results section, it is not clear to the reader if these C. elegans lines were generated in a previous study or created during the course of this work. If not generated in this study, the authors should add references for these lines.

>> The sources of all lines are indicated in Table S1, with the exception of the LGL-1::GFP which was generated as part of this work. We have added additional citations to manuscripts describing their creation in Table S1 where available. Unfortunately, the methods associated with some strains (notably KK lines) are not described in existing publications, but we have cited the first known instances.

On pg9, GUI should be defined, as it is the first time this term is used in the Manuscript.
>> Added.

In Fig 6 panel C, the arrows highlighting specific regions on the fluorescence linescans for the raw and SAIBR corrected samples are currently very difficult to see (especially for Nem1::mNG). The authors should consider alternatives ways to denote these regions.

>> The labeling in Figures 5 and 6 has been updated as suggested.

© 2022. Published by The Company of Biologists under the terms of the Creative Commons Attribution License (https://creativecommons.org/licenses/by/4.0/).

Second decision letter

MS ID#: DEVELOP/2022/200545

MS TITLE: SAIBR: A simple, platform-independent method for spectral autofluorescence correction

AUTHORS: Nelio T.L. Rodrigues, Tom Bland, Joana Borrego-Pinto, KangBo Ng, Nisha Hirani, Ying Gu, Sherman Foo, and Nathan W Goehring
ARTICLE TYPE: Techniques and Resources Article

I am happy to tell you that your manuscript has been accepted for publication in Development, pending our standard ethics checks.

Reviewer 1

Advance summary and potential significance to field

The authors have appropriately addressed my comments.

Comments for the author

This will be a very useful method!

Reviewer 2

Advance summary and potential significance to field

Fluorescence imaging is an important technique for observing the localization of specific proteins and monitoring biological processes in cells and tissues.
However, many organisms and cell types exhibit intrinsic autofluorescence (AF) that can obscure localization to specific structures and provides a significant barrier to downstream image analysis. Therefore, there is an unmet need for the development of user-friendly and easily implemented methods for excluding AF from microscopy images. Here Rodrigues et al. describe a new and straightforward protocol to correct for AF using standard filter sets and imaging conditions that enables accurate detection and quantitation of fluorescence signal. This intriguing study provides a new avenue to facilitate integration of AF correction as a standard part of imaging workflows.

Comments for the author

In the revised version of the manuscript “SAIBR: A simple, platform-independent method for spectral autofluorescence correction” by Nelio T.L. Rodrigues et al., the authors have taken steps to significantly address the suggestions of the reviewers. In my opinion, the authors have adequately addressed the reviewers' critiques, and the article is now suitable for publication in Development.

© 2022. Published by The Company of Biologists under the terms of the Creative Commons Attribution License (https://creativecommons.org/licenses/by/4.0/).
